# Serine ADPr on histones and PARP1 is a cellular target of ester-linked ubiquitylation

**DOI:** 10.1038/s41589-025-01974-5

**Published:** 2025-07-09

**Authors:** Andreas Kolvenbach, Maria Dilia Palumbieri, Thomas Colby, Diyaraj Nadarajan, Remo Bode, Ivan Matić

**Affiliations:** 1 Max Planck Institute for Biology of Ageing, Cologne, Germany; 2 Cologne Excellence Cluster for Aging and Aging-Associated Diseases (CECAD), University of Cologne, Cologne, Germany

**Keywords:** Proteomics, Post-translational modifications, Cell signalling, Mass spectrometry, Histone post-translational modifications

## Abstract

ADP-ribosylation and ubiquitylation regulate various cellular processes, with the complexity of their interplay becoming increasingly clear, as illustrated by ADP-ribosylation-dependent ubiquitylation mediated by *Legionella*. Biochemical studies have reported ester-linked ubiquitylation of ADP-ribose by DELTEX ubiquitin ligases, yet the modification sites on cellular targets remain unknown. Here, our search for interactors of RNF114 revealed DNA-damage-induced serine mono-ADP-ribosylation as a cellular target for ester-linked ubiquitylation. By developing proteomics strategies tailored to the chemical features of this composite modification, combined with an enrichment method using the zfDi19 and ubiquitin interaction motif domain (ZUD) of RNF114 and specific chemical elution, we identified ADP-ribosyl-linked serine ubiquitylation sites in cells, including on histones and poly(ADP-ribose) polymerase 1. Engineering ZUD into a modular reagent enabled the detection of this dual modification by immunoblotting. We establish ADP-ribosyl-ubiquitylation as an endogenous serine post-translational modification and propose that our multifaceted, tailored methodology will uncover its widespread occurrence, along with other conjugation chemistries, across diverse signaling pathways.

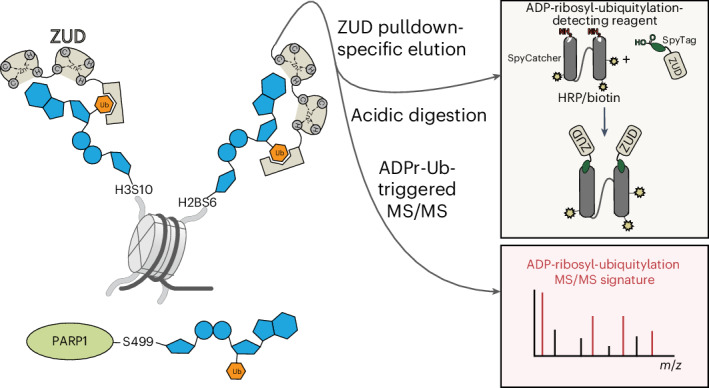

## Abstract (web summary)

RNF114 is an E3 ligase that can recognize ADP-ribose (ADPr) and ubiquitin with separate domains. Using these domains, Kolvenbach and Palumbieri et al. developed a proteomics approach to map ADP-ribosyl-ubiquitylation sites, revealing that serine ADPr induced by DNA damage is a target for ubiquitylation.

## Main

By greatly augmenting the functional versatility of the proteome, far beyond what is encoded by the genome, post-translational modifications (PTMs) are pivotal regulators of all signaling pathways^1^. Within complex and dynamic cellular networks, distinct PTMs often engage in crosstalk, occasionally interacting directly at the chemical level to generate composite signals. An emerging example of the combined chemistries of two distinct PTMs is the interplay between ADP-ribosylation (ADPr) and ubiquitylation^2^.

ADPr is a modification of protein, RNA and DNA that has crucial roles in regulating key biological functions across all forms of life^3^. Catalyzed by various ADP-ribosyltransferases, including poly(ADP-ribose) polymerases (PARPs), ADPr involves the covalent addition of ADP-ribose from nicotinamide adenine dinucleotide (NAD^+^) to diverse amino acid side chains and nucleotides through different conjugation chemistries, ranging from *O*-glycosidic to ester-linked ADPr^4,5^. This chemical diversity and versatility result in high complexity, leading to decades of technical challenges that have only recently been addressed through the development of effective tools and approaches^6,7^. Research on ADPr has primarily focused on PARP1’s role in the DNA damage response (DDR). PARP1 detects DNA breaks and modifies various targets, mainly itself and histones, to recruit DNA repair factors and chromatin remodelers to the DNA lesions^8^. Historically, poly-ADPr on aspartate and glutamate was considered as the only signal generated by PARP1 (refs. ^8–10^). Our initial identification of histone serine ADPr (Ser-ADPr)^11^ led us and others to establish Ser-ADPr by PARP1 and histone PARylation factor 1 (HPF1) as a widespread and functionally important PTM^12–17^. This discovery has prompted a profound reinterpretation of PARP1 signaling^18^, illustrating how uncovering the chemical nature of ADPr in cells can transform a research field. In a transient complex with HPF1 (ref. ^13^), PARP1 synthesizes mono-ADPr on serine residues^12^. In contrast, without HPF1, PARP1 extends the initial ADP-ribose to poly-ADPr^14,19^, forming the first wave of PARP1 signaling, and catalyzes mono-ADPr on aspartate and glutamate, which are the main target residues in cells lacking HPF1 (ref. ^20^). Several hydrolases remove different forms of ADPr. While ARH3 cleaves Ser-ADPr^21,22^, PARP glycohydrolase (PARG) breaks down poly-ADPr^23^ and, for some targets, especially PARP1 itself, acts as a poly-to-mono converting enzyme^24,25^. The inability of PARG to remove the initial ADP-ribose from serine residues^14^, combined with the PARP1–HPF1-mediated generation of mono-ADPr on these residues, gives rise to serine mono-ADPr^24^. This second wave of PARP1 signaling recruits the ubiquitin E3 ligase RNF114 to DNA lesions, where it regulates the DDR and telomere maintenance^24^.The recruitment of RNF114 to DNA lesions depends on its C-terminal zfDi19 domain and ubiquitin interaction motif (UIM), which recognize mono-ADPr and ubiquitin, respectively^24,26^. It has also been reported that RNF114 can recognize poly-ADPr^27^. Beyond its role in PARP1 signaling, RNF114 has recently been shown to recognize and stabilize mono-ADP-ribosylated tankyrase^28^.

In addition to functioning as a PTM on its own, ADPr can directly contribute to the conjugation chemistry of ubiquitylation^2^. A notable example of this interplay is a chemically unique form of ubiquitylation catalyzed by an effector of *Legionella pneumophila*, which ADP-ribosylates R42 of ubiquitin. This modification is then processed to enable conjugation of ubiquitin to serine residues through a phosphodiester bond, resulting in phosphoribose-linked serine ubiquitylation^29^. Another example is the unconventional ester-linked ubiquitylation of ADP-ribose on the 3′-hydroxy group of its adenine-proximal ribose, initially reported in studies using biochemical reactions with recombinant DELTEX ubiquitin E3 ligases^30^ and recently shown in cells through chemical and enzymatic removal approaches^31^. Given that DELTEX enzymes catalyze the ubiquitylation of free ADP-ribose and ADP-ribose attached to nucleic acid and proteins, as well as direct ubiquitylation of nucleic acids and canonical lysine-linked ubiquitylation^30,32–35^, it remains unclear which of these potential modifications occur in cells. Crucially, a series of technical challenges have hindered mass spectrometry (MS; the gold standard for PTM characterization) from detecting ester-linked ubiquitylation of ADP-ribose not only in cellular contexts but also in supposedly less challenging biochemical reactions.

Here, we overcome multiple challenges associated with proteomics analysis of ADP-ribosyl-ubiquitylation, providing the first identification of its modification sites. To this end, we combine our recently developed short, acidic ArgC digestion method with an enrichment strategy based on the zfDi19–UIM domain (ZUD) of RNF114. As a key step in the enrichment procedure, specific chemical elution is achieved through zinc ion chelation by EDTA, disrupting the binding of zfDi19 to mono-ADPr substrates. This biochemical enrichment strategy is complemented by proteomics approaches tailored to the unique chemical features of the composite PTM. Beyond proteomics, we applied the SpyTag protein ligation technology to convert ZUD into a reagent that enables the detection of cellular ADP-ribosyl-ubiquitylation by western blotting. Through these multilevel methodological advances, we unveiled ADP-ribosyl-linked serine ubiquitylation in cells within the context of PARP1 signaling, identifying sites on histones, PARP1 and additional targets. This discovery, along with our repertoire of approaches, paves the way for future identification of chemical variants of this composite PTM in diverse signaling pathways.

## Results

### Mono-ADPr-dependent interactome of RNF114

**Fig. 1:**
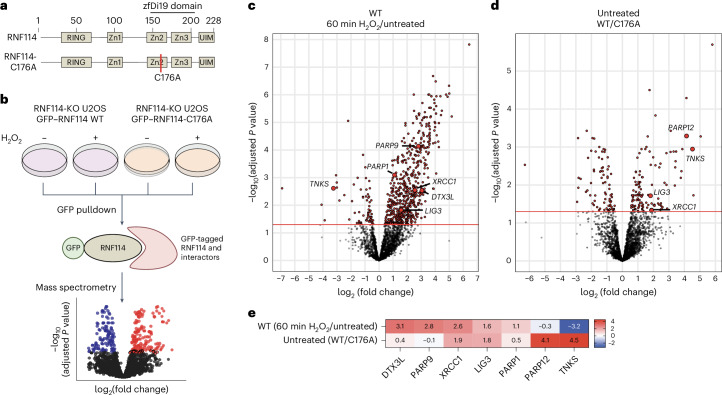
Identification of RNF114 interactors dependent on its zfDi19 domain and DNA damage. **a**, Schematic representation of RNF114 WT domain structure and C176A mutant. RNF114 contains a RING finger and Zn1 domain required for catalytic activity. Zn2 and Zn3 form the zfDi19 domain required for mono-ADPr binding. A single substitution in the zfDi19 domain (C176A) abolishes mono-ADPr binding. The UIM binds ubiquitin. **b**, Experimental setup of the GFP pulldown. RNF114-KO U2OS cells complemented with inducible GFP–RNF114 WT or GFP–RNF114-C716A were left untreated or treated with 1 mM H_2_O_2_ for 1 h. Each condition consisted of four biological replicates. The pulldown was performed under nondenaturing conditions to identify interactors dependent on an intact zfDi19 domain and DNA damage, followed by MS analysis. **c**, Volcano plot showing the log_2_ fold change of the interactors identified in RNF114 WT-overexpressing cells comparing the DNA-damage-treated and untreated conditions. **d**, Volcano plot showing the log_2_ fold change of the interactors identified under untreated conditions, comparing the cell lines overexpressing either GFP–RNF114 WT or GFP–RNF114-C176A. In **c**,**d**, statistical analysis was performed using limma’s two-sided moderated *t*-test. Adjusted *P* values were calculated using the Benjamini–Hochberg method to correct for multiple testing. The red dashed line in **c**,**d** displays significance with adjusted *P* values < 0.05 as the −log_10_(adjusted *P* value) > 1.3. **e**, Heat map showing the fold change of identified interactors from the comparisons shown in **c**,**d**.

**Extended Data Fig. 1:**
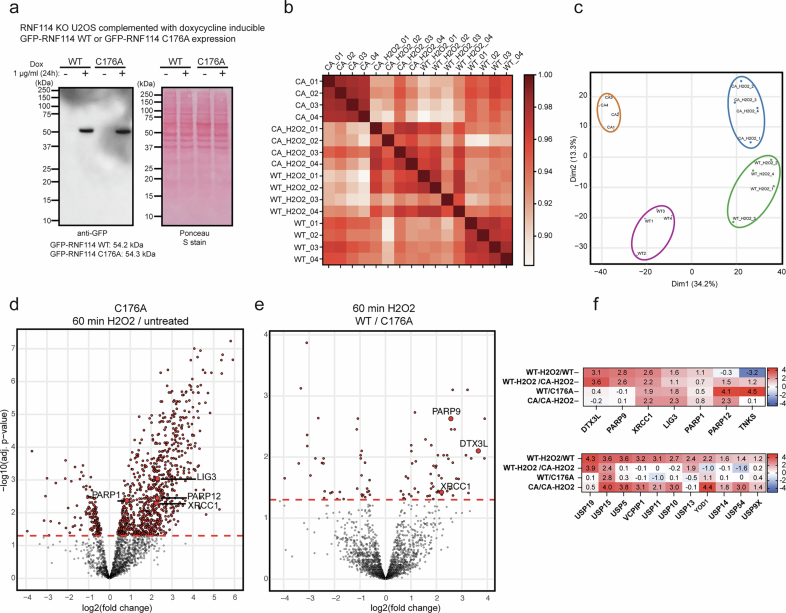
RNF114 interactors screen with GFP-RNF114 WT and C176A mutant inducible cell lines. **(a)** GFP immunoblot with and without 24-hour doxycycline induction in inducible RNF114 KO U2OS cells complemented with GFP-RNF114 WT or GFP-RNF114 C176A expression. **(b)** Correlation analysis output of DIA-NN, showing the sample correlation within and between the conditions. A general separation of the samples belonging to different conditions is visible. **(c)** PCA analysis confirming the clear separation of samples from different conditions during the GFP-Pulldown. **(d)** Volcano plot showing the log_2_-fold change of the interactors identified in H_2_O_2_-treated against untreated GFP-RNF114 C176A overexpressing cells. **(e)** Comparison of interactors in H_2_O_2_-treated cells either overexpressing GFP-RNF114 WT or GFP-RNF114 C176A. **(d,e)** The interactors stated in Fig. 1 are marked if significant. Statistical analysis was performed using the limma’s two-sided moderated t-test. Adjusted P values were calculated using the Benjamini-Hochberg method to correct for multiple testing. The red dashed line in **(d,e)** displays significance with adjusted P values < 0.05 as -log_10_(adj. P value) >1.3. **(f)** Heatmaps showing the log_2_-fold change of the interactors stated in Fig. 1 (above) and the log_2_-fold change of selected Deubiquitinases (below) across all comparisons. Source data

Recently, we showed that RNF114 binding to mono-ADPr depends on its zfDi19 zinc-binding domain, which is necessary for its recruitment to DNA lesions^24^. Considering that hundreds of proteins are mono-ADP-ribosylated upon DNA damage^12,15^, we sought to identify RNF114 interactors specifically dependent on its mono-ADPr-binding ability. To achieve this, we used the Sleeping Beauty transposon system to generate cell lines with inducible expression of GFP-tagged RNF114 wild type (WT) and GFP-tagged RNF114-C176A (Fig. 1a and Extended Data Fig. 1a). To identify ADPr-dependent binders of RNF114 in the context of DNA damage, we treated cells with H_2_O_2_ for 60 min, a condition that maximizes the induction of serine mono-ADPr^24^, and performed the GFP pulldown followed by data-independent acquisition (DIA) quantitative proteomics analysis (Fig. 1b, Extended Data Fig. 1 and Supplementary Data 1). As we previously reported^24^, the substitution of C176 completely abolishes the ability of RNF114 to bind mono-ADPr. Thus, comparing RNF114 WT and RNF114-C176A, as well as untreated and H_2_O_2_-treated conditions (Fig. 1), enabled the specific identification of interactors dependent on its ADPr-binding ability and DNA damage. Among the identified interactors, we detected several DNA repair proteins, including PARP1, XRCC1 and LIG3, as well as the E3 ubiquitin ligase DTX3L (Fig. 1c,e). Interestingly, in the absence of DNA damage, we also identified the ADP-ribosyltransferases PARP12 and tankyrase, suggesting that RNF114 binds ADPr targets beyond the DDR, as previously shown for tankyrase^28^ (Fig. 1d,e). Additionally, we detected several deubiquitinases (Extended Data Fig. 1f), some of which may act as erasers of ubiquitin from ADP-ribose.

### Identification of peptides comodified by ubiquitin and ADPr

**Fig. 2:**
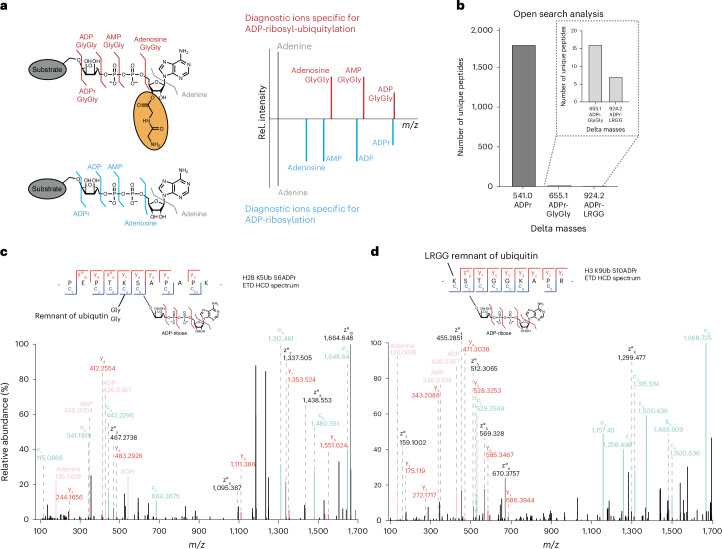
Analysis of published datasets for combinations of mono-ADPr and ubiquitylation. **a**, Expected fragmentation pattern of ADP-ribosyl-ubiquitylation and mono-ADPr. ADP-ribosyl-ubiquitylation results in specific diagnostic ions (red), including ADP-GlyGly, AMP-GlyGly, adenosine-GlyGly and adenine. Conventional mono-ADPr on substrates with or without other modifications results in a well-known set of diagnostic ions emerging from mono-ADPr (blue), consisting of complete mono-ADPr, ADP, AMP, adenosine and adenine. The two sets of diagnostic ions share adenine as a diagnostic ion (gray). The masses of ADP-GlyGly and mono-ADPr differ only slightly, while adenosine-GlyGly and AMP-GlyGly can be clearly distinguished from AMP and ADP of conventional mono-ADPr. **b**, Results of an open search of the dataset PXD023835, searching for masses combining mono-ADPr (541.0611 Da) and ubiquitylation after digestion (GlyGly, 114.0429 Da; LRGG, 383.2281 Da). The open search results were filtered using less stringent mass tolerances to account for potential mass errors. The number of unique peptides identified for each delta mass (mono-ADPr, 541.1 Da; ADPr-GlyGly, 655.1 Da; ADPr-LRGG, 924.2 Da) is displayed. Inset, zoomed-in view to better visualize the lower-abundance delta masses (655.1 and 924.2 Da). **c**, Identified spectra of neighboring ubiquitylation (GlyGly) on K5 and mono-ADPr on S6 of H2B. **d**, Identified spectra of neighboring ubiquitylation (LRGG) on K9 and mono-ADPr on S10 of H3. The spectra in **c**,**d** display the conventional mono-ADPr diagnostic ions, indicating the presence of unmodified mono-ADPr.

Having identified mono-ADPr targets recognized by RNF114, we aimed to investigate the molecular basis of this interaction by pinpointing the specific modification sites read by this ubiquitin ligase. While we showed that the zfDi19 domain of RNF114 is necessary for binding in vitro to a mono-ADP-ribosylated peptide^24^, a UIM domain is also required for the recruitment of RNF114 to DNA lesions^26^. This suggests that RNF114 functions as a reader of both mono-ADPr and ubiquitylation, motivating us to develop a computational proteomics approach capable of distinguishing between the various possible ubiquitylation/ADPr chemistries. Our identification of DTX3L in RNF114 interactome (Fig. 1) and recruitment of DTX3L to serine mono-ADPr upon DNA damage^24^ suggest that RNF114 may act as a reader of ester-linked ubiquitylation of ADP-ribose, catalyzed in vitro by DTX ligases. However, we sought an approach that also considers other potential dual ADP-ribose and ubiquitin signals, including ADPr of ubiquitin and separate modifications occurring in close proximity (Fig. 2a). This differs from conventional proteomics data analysis, which makes it difficult to identify and distinguish between different possible composite ubiquitin–ADPr chemistries.

The first step of our computational proteomics approach is an open search to determine clusters of delta masses, followed by the inspection of diagnostic ions in higher-energy collision dissociation (HCD) fragmentation spectra. The presence of standard ADP-ribose-derived diagnostic ions indicates that any additional peptide modification is on the peptide backbone rather than on ADP-ribose itself. In contrast, if these diagnostic ions show mass shifts, potentially because of the GlyGly remnant from digested ubiquitin, it indicates that ADP-ribose is the direct target of the additional modification (Fig. 2a). Given the availability of several large-scale ADPr proteomics studies and the successful application of our reanalysis approaches to uncover hidden forms of ADPr^12,36,37^, we reasoned that we could obtain cellular evidence of ubiquitylation and ADPr through our tailored reanalysis strategy. Intriguingly, through open search analysis (Fig. 2b and Supplementary Data 1) and inspection of electron-transfer dissociation (ETD) and HCD MS/MS spectra^38^, we discovered that histone peptides are comodified by ubiquitin on the lysine immediately preceding the ADP-ribosylated serine (Fig. 2c,d), suggesting an interplay that could be as important as the crosstalk observed with histone phosphorylation, acetylation and ADPr^36^. Despite the two modifications occurring on adjacent amino acids, the exclusive detection of ADP-ribose-specific diagnostic ions alongside the peptide backbone fragment ions clearly indicates that ubiquitylation is a separate modification occurring on the preceding lysine. We did not find evidence of ubiquitylated ADP-ribose in our reanalysis of published datasets (Supplementary Fig. 1). This is expected, given the high chemical lability of the ester bond linking ubiquitin to ADP-ribose. The reanalyzed published datasets rely on experimental conditions, such as high pH for protein digestion and peptide fractionation, which cleave ester bonds^20,39^. In fact, these studies have identified a negligible number of ester-linked aspartate and glutamate ADPr sites^15,38,40^, further underscoring the general inadequacy of standard proteomics approaches for ester-linked modifications.

### Identification of ADP-ribosyl-ubiquitylation sites using ZUD

While our computational proteomics approach can identify and distinguish between different combinations of ubiquitin and ADPr (Fig. 2), ester-linked modifications, including ubiquitylation of ADP-ribose, are largely lost in conventional proteomics preparation procedures^20,39^. Therefore, specialized sample preparation and pulldown protocols are essential to preserve, enrich and identify the exact ADPr–ubiquitin sites recognized by RNF114. To this end, we combined our recently introduced methods for preserving ester-linked modifications^20^ with the development of a specific enrichment strategy.

**Fig. 3:**
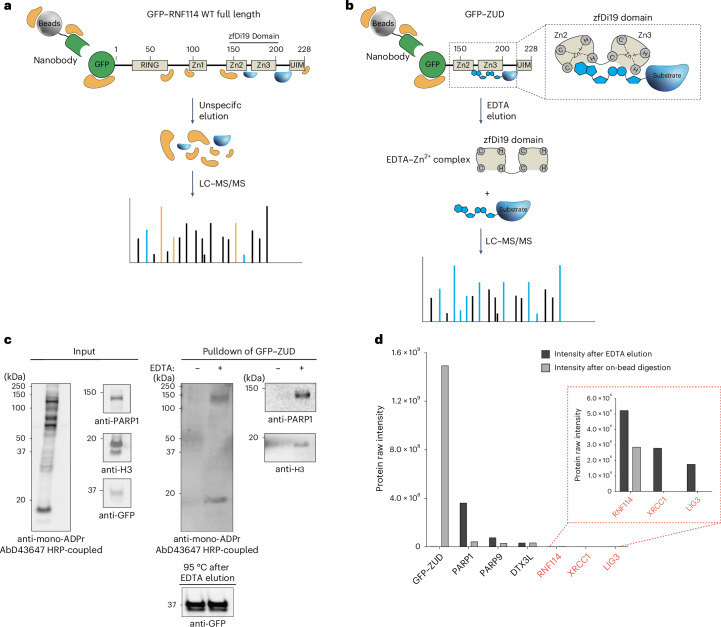
ZUD enrichment and EDTA elution strategy. **a**, Scheme of the full-length GFP–RNF114 WT pulldown, illustrating that this approach also enriches proteins that bind nonspecifically to the beads, the GFP tag and other domains of RNF114, potentially complicating downstream analysis. **b**, Performing the GFP pulldown with ZUD focuses the pulldown on interactors of the two domains of RNF114 but still coenriches unspecific binders of the beads and the GFP tag. To minimize this, an EDTA elution is used to remove Zn²⁺ ions from the zfDi19 domain, releasing ADP-ribosylated proteins and proteins carrying ADP-ribosyl-ubiquitylation, simplifying the MS/MS analysis. **c**, GFP–ZUD pulldown of ARH3-KO cells (one 15-cm dish) transfected with GFP–ZUD and treated with 2 mM H_2_O_2_ (30 min). EDTA-specific elution (5 min at 37 °C) elutes mono-ADPr proteins (AbD43647–HRP-coupled blot), as well as PARP1 and histone H3. Following the EDTA elution, GFP–ZUD was eluted by heating the beads at 95 °C for 15 min. Shown is a representative result from three independent experiments. **d**, Comparison of protein intensities during MS/MS analysis after digestion of the elution and subsequent on-bead digestion of the eluted beads. Two 500-cm^2^ dishes of ARH3-KO cells transfected with GFP–ZUD were treated with 2 mM H_2_O_2_ (30 min). EDTA elution: 15 min at 37 °C. The results clearly show that the bait remains bound to the beads, while PARP1, XRCC1 and LIG3 are specifically released by EDTA. Inset, zoomed-in view highlighting the lower-intensity proteins (RNF114, XRCC1 and LIG3) for better visualization. Source data

In general, identifying PTM sites in complex peptide mixtures (for example, from whole-cell extracts or insufficiently enriched samples) is much more challenging than detecting substrate proteins. In fact, the on-bead digestion protocol that enabled the identification of RNF114 interactors (Fig. 1) did not yield any ADPr sites. For this reason, we first switched from full-length GFP–RNF114 to GFP–ZUD to focus our proteomics analyses on the interactors of the C-terminal region of RNF114 (Fig. 3a,b). Second, we reasoned that an elution approach preventing the elution of the bait would increase the likelihood of successfully identifying the modification sites (Fig. 3b).

**Extended Data Fig. 2:**
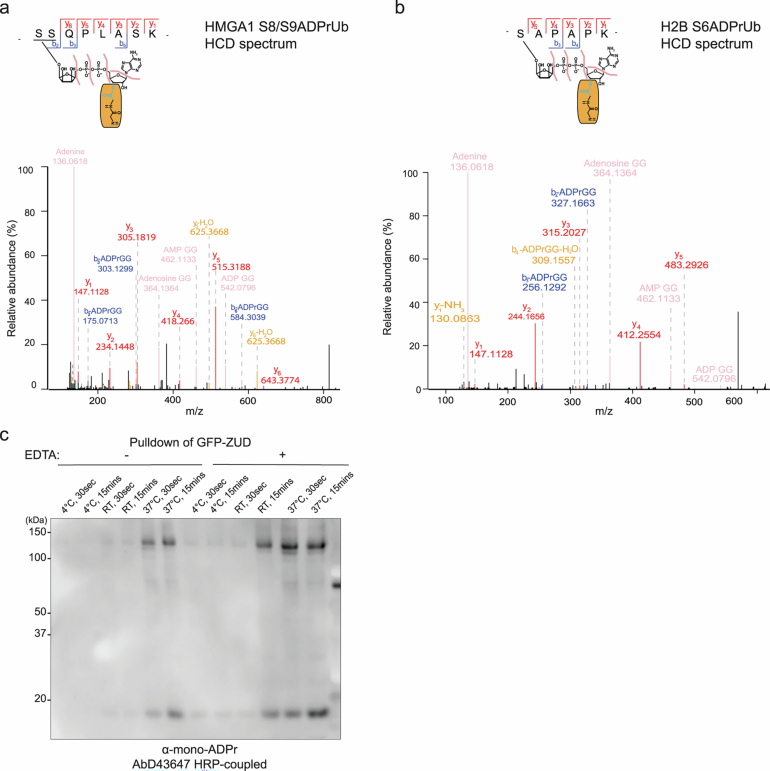
Initial identification of ADP-ribosyl-ubiquitylation sites and EDTA elution conditions. **(a)** HCD spectrum of ADP-ribosyl-ubiquitylation identified on HMGA1. **(b)** HCD spectrum of H2B showing the diagnostic ions of ADP-ribosyl-ubiquitylation. **(a,b)** Elution: 8 M Urea for 10 min at 37 °C. Four 10 cm dishes of U2OS WT cells transfected with GFP-ZUD were treated with 2 mM H_2_O_2_ (30 min). **(c)** Optimization of the EDTA elution temperature (4 °C, RT and 37 °C) and timing (30 seconds and 15 min). Shown is a representative result from three independent experiments. Source data

Hoping to exploit the stability of GFP and its strong binding to nanobody-coupled beads, we tested elution with 8 M urea^20^, which leaves the abundant GFP–ZUD bait on the beads while eluting both specific and nonspecific binders. To preserve the ester bond linking ubiquitin to ADP-ribose, we applied our short acidic digestion protocol with ArgC and LysC proteases^20^ to the eluted proteins. Notably, alongside conventional serine mono-ADPr sites, we observed HCD MS/MS spectra containing adenine but with further diagnostic fragment ions shifted by a GlyGly mass, the typical ubiquitin signature that remains after tryptic digestion. These ions appear consistent with a form of ADP-ribose ubiquitylated on its adenine-proximal ribose; thus, these spectra provide evidence of ADP-ribosyl-ubiquitylation sites (Fig. 2). This fragment ion signature appears specific for ubiquitylated ADP-ribose: ADP-GlyGly, AMP-GlyGly and adenosine-GlyGly (Fig. 2a). By including these masses in a MaxQuant search, we achieved the initial identification of HMGA1 and H2B peptides as targets of ADP-ribosyl-linked serine ubiquitylation in WT U2OS cells (Extended Data Fig. 2a,b). This result encouraged us to pursue a more specific elution method to prevent the elution of both bait and nonspecific binders. Because RNF114 recognizes mono-ADPr through the two zinc fingers of its zfDi19 domain, we reasoned that sequestration of zinc ions by EDTA (previously shown to disrupt the interaction between recombinant RNF114 and a mono-ADPr peptide^24^) might specifically elute mono-ADPr targets from zfDi19 and, consequently, from ZUD (Fig. 3b). This disruption of the structural integrity of the zfDi19 zinc fingers reduces the elution of the abundant bait and nonspecific binders, thereby focusing the proteomic analyses on proteins carrying the modification. By western blotting, we confirmed that EDTA specifically elutes mono-ADPr targets from GFP–ZUD beads (Fig. 3c and Extended Data Fig. 2c). As anticipated, MS analysis showed that, compared to on-bead digestion, this specific elution method focused the MS analysis on RNF114 interactors (Fig. 3d).

Alkylation, a common step in protein digestion protocols, is a potential artifact that can mimic ubiquitin attachment to lysine residues in proteomics analyses^41^. While hydroxyl groups, including the hydroxyl of the adenine-proximal ribose targeted by ester-linked ubiquitylation, have not been reported to form alkylation artifacts, we used chloroacetamide, which effectively prevents such artifacts compared to the traditionally used iodoacetamide^41^. To completely exclude the possibility that our identification of GlyGly-modified ADP-ribose is because of an alkylation artifact, we incubated a serine mono-ADP-ribosylated peptide with chloroacetamide, following the same protocol used for protein digestion. We detected the mono-ADPr peptide and a very minor amount of peptide with a mass shift potentially corresponding to single alkylation, a modification that does not mimic the GlyGly remnant of ubiquitin. Importantly, while we detected many spectra with the standard diagnostic ions of ADPr, we found no evidence of spectra containing the diagnostic ion pattern specific to ADP-ribosyl-linked ubiquitylation—the key criterion introduced here for confidently identifying this composite modification (Supplementary Fig. 2). This confirms that alkylation was not responsible for the modification corresponding to the mass of GlyGly on ADP-ribose observed on target peptides.

### A double-triggering MS strategy

While we were able to uncover the first sites of this composite modification in WT cells (Extended Data Fig. 2a,b), the abundance of these peptides remained low compared to the predominance of unmodified contaminating peptides, hindering MS analysis of this composite PTM. Proteomics of simpler PTMs typically involves enriching modified peptides and depleting those peptides that do not carry the modification of interest, including those from target proteins. However, because UIM motifs interact with the hydrophobic patch of intact, undigested ubiquitin^42^, peptide-level enrichment cannot be used with ZUD. Thus, while EDTA elution greatly simplifies the sample at the protein level, the peptide mixture remains complex. Furthermore, the potential partial cleavage of the ester bond may further decrease the relative abundance of modified peptides, despite our efforts to preserve it. To improve our means of detecting ADP-ribosyl-linked ubiquitylation within this remaining complexity, we developed strategies to focus MS more precisely on this composite PTM.

**Fig. 4:**
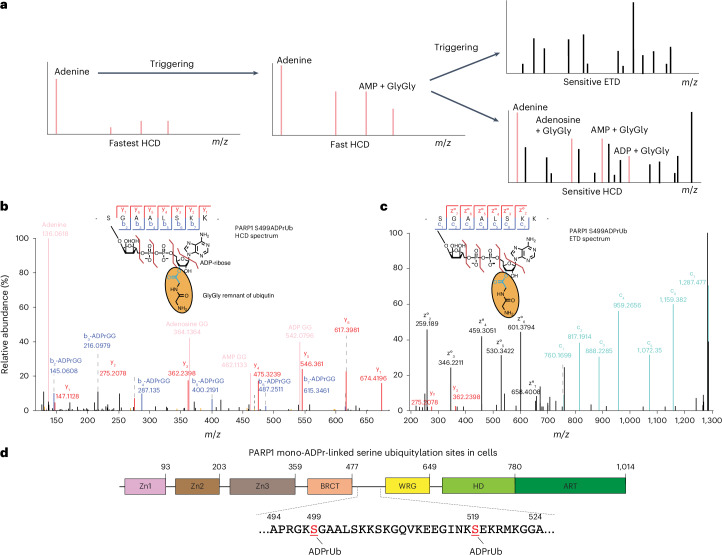
Optimized MS methods enable confident localization of ADP-ribosyl-ubiquitylation using ETD and HCD fragmentation. **a**, Detection workflow for ADP-ribosyl-ubiquitylation. This approach relies on triggering a medium-quality HCD scan upon detecting *m*/*z* corresponding to Adenine. If the subsequent triggered MS2 scan reveals *m*/*z* corresponding to AMP-GlyGly or adenosine-GlyGly, it triggers ETD or HCD fragmentation, depending on the chosen method. **b**,**c**, HCD and ETD spectra containing ADPr-GlyGly diagnostic ions and localizing ADP-ribosyl-ubiquitylation to S499 of PARP1. Two 500-cm^2^ dishes of ARH3-KO cells transfected with GFP–ZUD were treated with 2 mM H_2_O_2_ (30 min). EDTA elution was performed for 15 min at 37 °C.

**Fig. 5:**
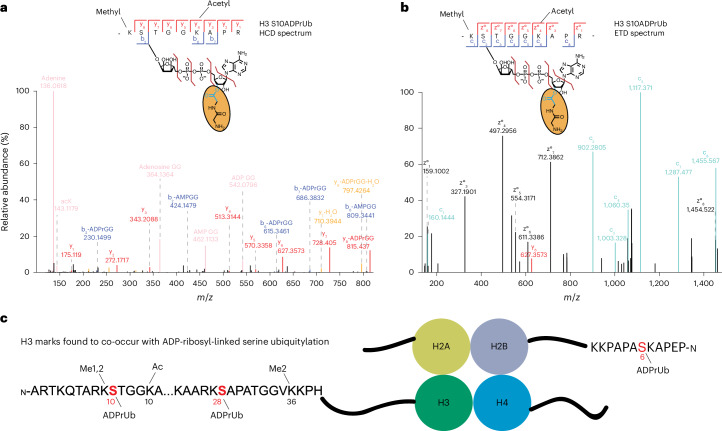
Histone ADP-ribosyl-ubiquitylation marks. **a**,**b**, HCD and ETD spectra containing ADPr-GlyGly diagnostic ions and localizing ADP-ribosyl-ubiquitylation to S10 of H3. Three 500-cm^2^ dishes of ARH3-KO cells transfected with GFP–ZUD were treated with 2 mM H_2_O_2_ (30 min). EDTA elution was performed for 20 min (**a**) and 5 min (**b**) at room temperature with ArgC digested in **a**. **c**, Schematic of the nucleosome structure depicts histones H2A, H2B, H3 and H4 and the ADP-ribosyl-ubiquitylation sites identified in cells on histones H3 and H2B. H3 is modified with ADP-ribosyl-ubiquitylation on S10 and S28. Additional histone marks such as methylation (Me1 and Me2) and acetylation (Ac) are indicated. H2B is modified with ADP-ribosyl-ubiquitylation on S6. Modified serine residues are shown in red.

**Extended Data Fig. 3:**
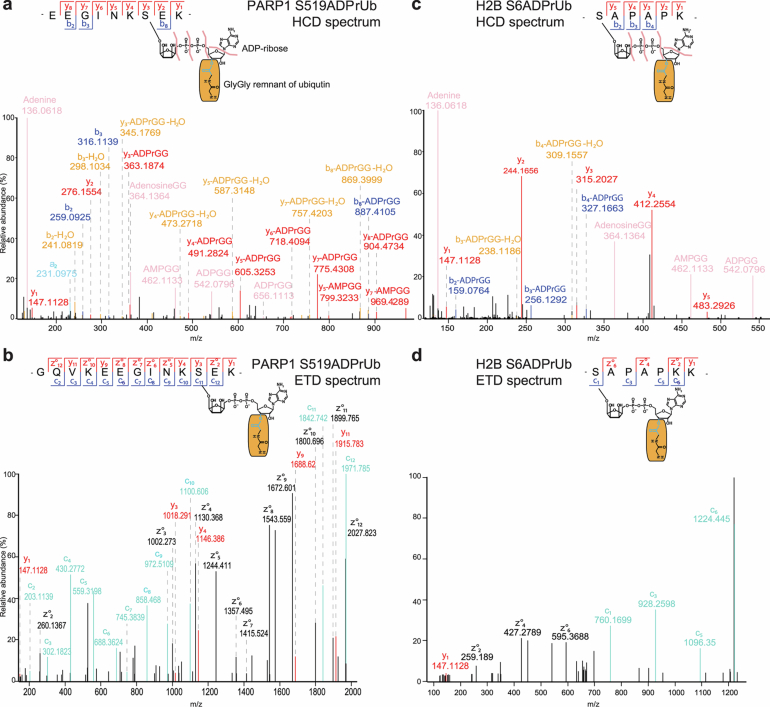
Additional spectra of peptides modified with ADP-ribosyl-ubiquitylation. **a,b)** HCD and ETD spectra of ADP-ribosyl-ubiquitylation on serine 519 of PARP1. **(c,d)** HCD and ETD spectra of ADP-ribosyl-ubiquitylation on serine 6 of H2B. All shown HCD spectra contain the diagnostic ions of ADP-ribosyl-ubiquitylation. The ETD spectra do not contain diagnostic ions as the modification stays intact and is not fragmented, thus allowing the localization of the modification site. These spectra illustrate that ETD data is critical to localize the ADP-ribosyl-ubiquitylation on peptides. However, the presence of ADP-ribosyl-ubiquitylation can be validated by diagnostic ions using HCD. This illustrates how HCD and ETD data can complement each other. **(a-d)** EDTA elution: 15 min at 37 °C. Two 500-cm^2^ dishes of ARH3 KO cells transfected with GFP-ZUD were treated with 2 mM H_2_O_2_ (30 min).

**Extended Data Fig. 4:**
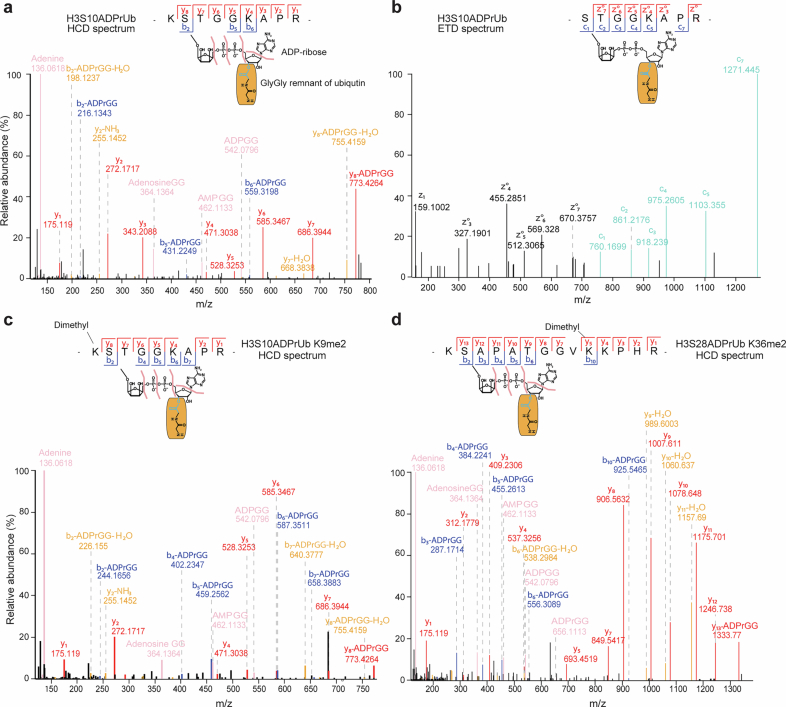
Additional spectra of histone ADP-ribosyl-ubiquitylation marks. **(a,b)** HCD and ETD spectra of ADP-ribosyl-ubiquitylation on serine 10 of H3. **(c,d)** HCD spectra of ADP-ribosyl-ubiquitylation likely on H3 serine 10 **(c)** or serine 28 **(d)** co-occurring with di-methylation of lysine 9 and lysine 36, respectively. **(b)** EDTA elution: 15 min at 37 °C. ARH3 KO cells (two 500-cm^2^ dishes) transfected with GFP-ZUD were treated with 2 mM H_2_O_2_ (30 min). **(a,c,d)** EDTA elution: 20 min at RT and ArgC digested. ARH3 KO cells (three 500-cm^2^ dishes) transfected with GFP-ZUD were treated with 2 mM H_2_O_2_ (30 min).

Building on our successful adenine-triggering to obtain high-quality HCD and ETD spectra^24,25^, we designed a more sensitive and specific MS approach. This involves first detecting adenine in fast, low-quality HCD spectra to trigger acquisition of medium-quality HCD spectra. Detection of either ADPr-GlyGly or AMP-GlyGly in these spectra triggers high-quality MS2 (Fig. 4a). Moreover, to improve the detectability of ADP-ribosyl-linked serine ubiquitylation by increasing its cellular levels, we also used ARH3-knockout (KO) cells^21^, as the absence of this hydrolase elevates serine mono-ADPr levels and prolongs RNF114 recruitment at DNA lesions^24,25^. This approach enabled the acquisition of high-quality ETD and HCD spectra, leading to confident identification of ADP-ribosyl-linked ubiquitylation on PARP1, histones H3 and H2B, HMGA1 and HNRNPU (Figs. 4b–d and 5a–c, Extended Data Figs. 3 and 4, Supplementary Fig. 3 and Supplementary Data 1). Importantly, the presence of several prominent specific diagnostic ions clearly rules out the possibility that ADPr and ubiquitylation co-occur on different residues of the target peptide. In such a case, the HCD spectra would contain exclusively standard ADP-ribose diagnostic ions, as shown above (Fig. 2). Specifically, for ADP-ribosyl-ubiquitylation, we observed ions corresponding to singly charged adenine (*m*/*z* 136.061), adenosine with GlyGly (*m*/*z* 364.136), AMP with GlyGly (*m*/*z* 462.113) and ADP with GlyGly (*m*/*z* 542.079). In contrast, conventional serine mono-ADPr yields diagnostic ions of adenine (*m*/*z* 136.061), AMP (*m*/*z* 348.068), ADP (*m*/*z* 428.034) and full ADP-ribose (*m*/*z* 542.068) (Fig. 2a). While ADP-GlyGly and unmodified ADP-ribose are nearly identical (*m*/*z* 542.079 and *m*/*z* 542.068), the presence of AMP with GlyGly and Adenosine with GlyGly clearly distinguishes ADP-ribosyl-ubiquitylation from conventional ADPr (Supplementary Fig. 4). Furthermore, high-resolution ETD, which does not fragment the modification^11^, confirmed site identification by confidently pinpointing the exact modified residue. Following MaxQuant^43^ analysis, we manually inspected and annotated all the representative MS/MS spectra (Figs. 4b,c and 5a,b, Extended Data Figs. 3 and 4 and Supplementary Fig. 3). All the reported peptides are confidently identified as modified by GlyGly-ADP-ribose on the basis of the presence of multiple diagnostic ions in the HCD spectra. ETD spectra precisely localized the composite PTM to S499 and S519 of PARP1 (Fig. 4b,c and Extended Data Fig. 3a,b), H3S10 (Fig. 5a,b, Extended Data Fig. 4a–c and Supplementary Fig. 3c,d) and H2BS6 (Extended Data Fig. 3c,d). However, pinpointing the modification site for HMGA1, HNRNPU and H3S28 (also confident targets of ADP-ribosyl-linked ubiquitylation) was less definitive, as we obtained only spectra with HCD fragmentation (Supplementary Fig. 3a,b and Extended Data Fig. 4d), which leads to Ser-ADPr lability^44^. Nevertheless, serines on all identified peptides are established sites of HPF1–PARP1-dependent ADPr^11,12,15,25,40,45^. Similarly to our analysis of the interplay between conventional Ser-ADPr and other histone marks^11,36^, we identified the ADP-ribosyl-ubiquitylated H3S10 and H3S28 in combination to additional histone marks. This includes monomethylation and dimethylation on K9 and acetylation of K14 in the vicinity of ADP-ribosyl-ubiquitylated S10 (Fig. 5a,b, Extended Data Fig. 4c and Supplementary Fig. 3c,d). Interestingly, the ADP-ribosyl-ubiquitylated peptide with the H3S28 site was identified only when co-occurring with K36 dimethylation (Extended Data Fig. 4d).

**Extended Data Fig. 5:**
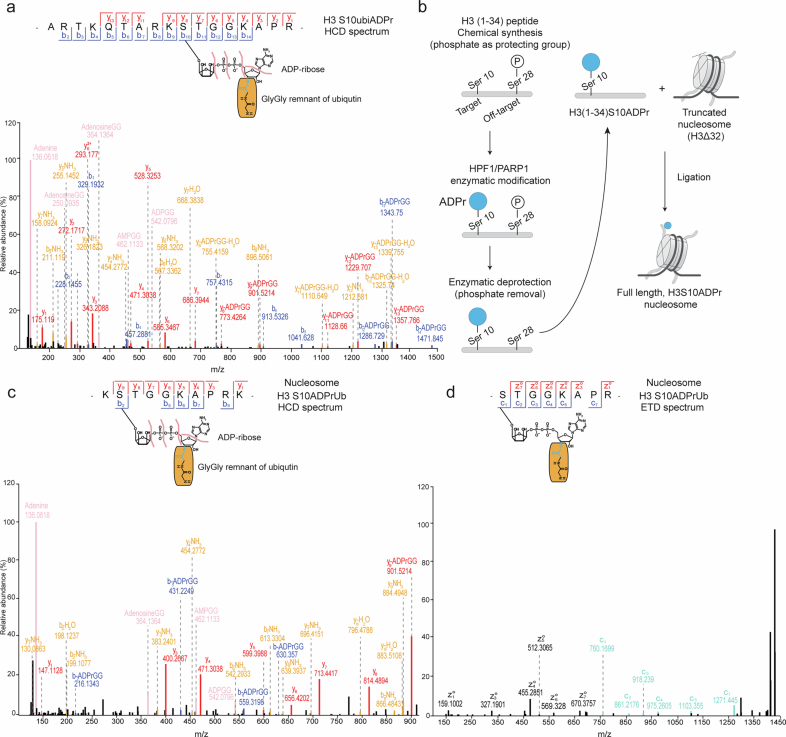
*In vitro* reactions using DTX2 and DTX3L to catalyze ADP-ribosyl-ubiquitylation on H3S10ADPr peptide or nucleosome. **(a)** HCD spectra of the *in vitro* reaction with DTX2 RING-DTC domains and biotinylated ADP-ribosylated H3 peptide modified with ADP-ribosyl-ubiquitylation on serine 10. **(b)** Production of site-specific H3S10ADPr nucleosomes (adapted from and described in^24^). **(c)** HCD and **(d)** ETD spectra of the *in vitro* reaction with DTX3L RING-DTC domains and biotinylated ADP-ribosylated H3 nucleosomes modified with ADP-ribosyl-ubquitylation on serine 10.

Using our serine mono-ADPr peptide^25^, we reproduced the H3S10 site identified in cells in an in vitro DTX2 ubiquitylation reaction (Extended Data Fig. 5a). To further corroborate ADP-ribosyl-ubiquitylation of H3S10 in a more physiological context, we performed the DTX3L reaction on an H3S10ADPr nucleosome. This nucleosome was generated using our phospho-guided strategy^25^ to site-specifically ADP-ribosylate the H3 tail, followed by ligation to a fully assembled nucleosome with tailless H3 (ref. ^24^) (Extended Data Fig. 5b–d). These MS identifications of the same site found in cells, indicate that DELTEX enzymes catalyze this histone mark.

### Conversion of ZUD into a modular reagent

Recently, we advanced mono-ADPr detection by integrating the SpyTag–SpyCatcher protein ligation technology^46^ into our recombinant antibodies^24,25^, enabling the rapid and straightforward coupling of Fab antibodies to enzymes, probes and Fc regions^24,47^. Inspired by this approach and the Kraus laboratory’s conversion of ADPr-binding domains into detection reagents^48^, we reasoned that combining ZUD recognition of ADP-ribosyl-ubiquitylation with SpyTag technology could generate a versatile research tool. The concept of ZUD as a detection reagent for this dual modification originates from our successful pulldown and identification of ADP-ribosyl-ubiquitylation sites (Figs. 3–5) and is further supported by the detailed characterization of its binding properties^49^.

**Fig. 6:**
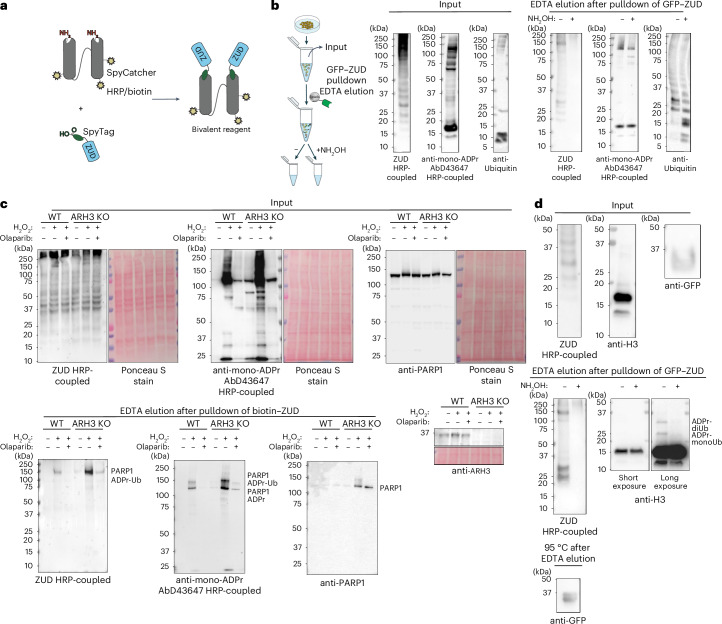
Converting ZUD into a detection reagent using the SpyTag–SpyCatcher technology. **a**, The SpyTag–SpyCatcher technology enables spontaneous isopeptide bond formation of the amino group of lysine side chains and the carboxyl group of aspartate side chains. This allows Spy-tagged proteins such as ZUD to be coupled to HRP for immunoblotting, Fc for immunofluorescence and biotin for streptavidin-based enrichment (Extended Data Fig. 6a). **b**, GFP–ZUD pulldown of ARH3-KO cells (four 500-cm^2^ dishes) transfected with GFP–ZUD and treated with 2 mM H_2_O_2_ (30 min). One sample was processed and the elution was split in two as described in the scheme (top). One half was treated with 1 M hydroxylamine (NH₂OH) and the other half was left untreated (−). The ZUD–HRP signal is abolished after treating the elution with NH₂OH for 2 h. This treatment preserves the mono-ADPr pattern (AbD43647–HRP-coupled) and the overall ubiquitin signal. Shown is a representative result from three independent experiments. **c**, Streptavidin pulldown of biotin-coupled ZUD from untreated, DNA-damage-treated (2 mM H_2_O_2_, 30 min) or DNA-damage-treated and olaparib-treated (1 µM) untransfected U2OS WT and untransfected ARH3-KO cells (two 15-cm dishes per condition were used). Immunoblots of the elutions for ZUD–HRP, AbD43647–HRP-coupled and PARP1 antibodies reveal a DNA-damage-dependent increase in ZUD–HRP signal and in mono-ADPr and PARP1. The bands likely to correspond to PARP1, PARP1 ADPr and PARP1 ADPrUb are labeled on each elution blot. An 8% Bis–Tris gel was used for the anti-PARP1 and AbD43647–HRP-coupled blots. A 4–12% Bis–Tris gradient gel was used for the ZUD–HRP-coupled blot. Shown is a representative result from three independent experiments. **d**, GFP–ZUD pulldown of ARH3-KO cells (eight 500-cm^2^ dishes) transfected with GFP–ZUD and treated with 2 mM H_2_O_2_ (30 min). The elution was split in two and half of it was treated with hydroxylamine (NH₂OH) as described in **b**. The H3 blot reveals NH_2_OH-sensitive ADPr-monoUb and ADPr-diUb bands. A 20% Bis–Tris gel was used to better resolve H3. A 4–12% Bis–Tris gradient gel was used for the ZUD–HRP-coupled blot. Following the EDTA elution, GFP–ZUD was eluted by heating the beads at 95 °C for 15 min. Shown is a representative result from four independent experiments. In **b**–**d**, EDTA elution was performed for 5 min at room temperature. Source data

**Extended Data Fig. 6:**
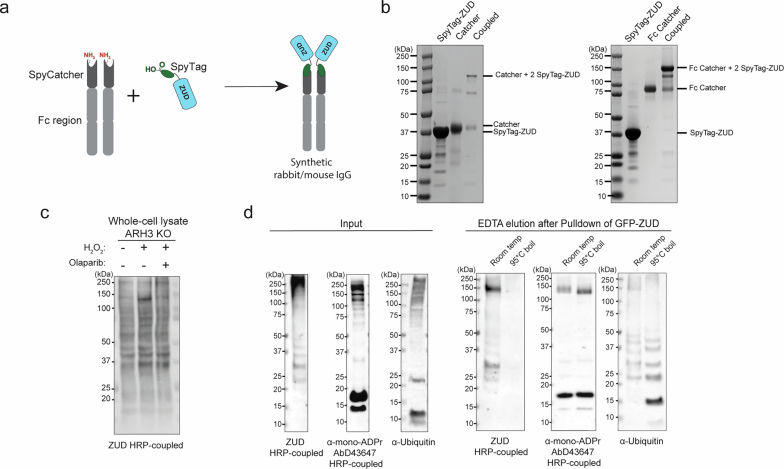
Coupling of SpyCatcher in different formats to the SpyTag-ZUD and specificity of ZUD. **(a)** Scheme of the SpyCatcher-SpyTag reaction and the bivalent antibody-like reagent. **(b)** The coupling was analysed by Coomassie staining. On the left, the Biotin SpyCatcher coupling to the SpyTag-ZUD is shown. On the right, the Fc SpyCatcher coupling to the SpyTag-ZUD. Both gels display the uncoupled ZUD (SpyTag-ZUD) as input, the respective uncoupled SpyCatcher (Catcher), and the result of the coupling (coupled). 2 µg of each sample were loaded. **(c)** Signal of HRP-ZUD reagent in whole cell lysates of untreated, H_2_O_2_-treated and Olaparib + H_2_O_2_-treated ARH3 KO cells from a replicate of the experiment shown in Fig. 5c. Shown is a representative result from at least three independent experiments. **(d)** GFP-ZUD pulldown of ARH3 KO cells (four 500 cm^2^ dishes) transfected with GFP-ZUD and treated with 2 mM H_2_O_2_ (30 min). Half of the EDTA elution (5 min RT) was heated to 95 °C for 5 min. The ZUD-HRP signal is abolished after boiling. This treatment preserves the mono-ADPr pattern (AbD43647 HRP-coupled) and the overall ubiquitin signal. Shown is a representative result from three independent experiments. Source data

Applying the SpyTag technology to ZUD enables the generation of dimeric ZUD reagents, mimicking the bivalent nature of standard immunoglobulins (Fig. 6a). The resulting avidity effect, arising from the synergistic binding of two ZUD copies, increases sensitivity, as demonstrated for recombinant Fab antibodies^24,47^. Additionally, immunoblotting sensitivity can be further increased with a specialized format that is site-specifically coupled to three horseradish peroxidase (HRP) moieties^24,47^. Moreover, SpyTag–ZUD can be adapted to other bivalent formats, including biotin-coupled tools and, through the addition of Fc regions, human, rabbit and mouse IgG-like reagents^47^ (Fig. 6a and Extended Data Fig. 6a). Compared to standard IgG or IgG-like antibodies, the HRP format is more sensitive^20,24,47^ but limited to immunoblotting. In contrast, adding an Fc region creates a more versatile bivalent reagent that is recognizable by standard secondary antibodies.

**Extended Data Fig. 7:**
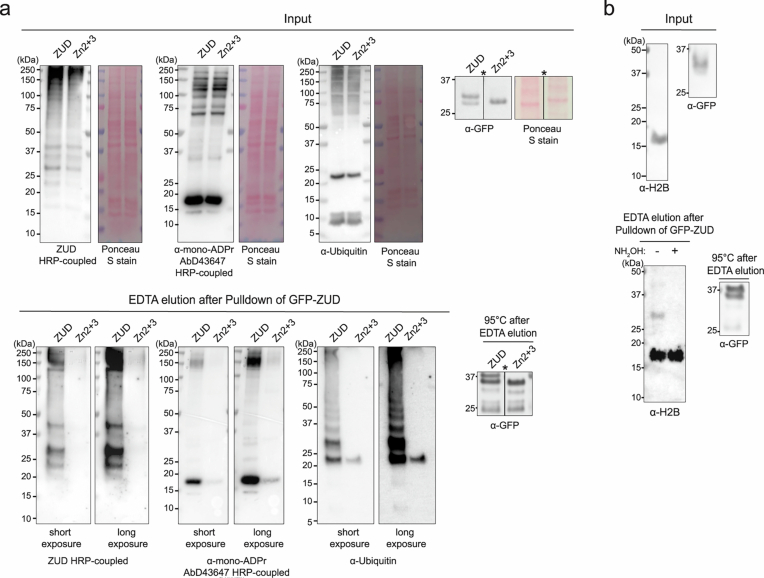
Specificity of ZUD and pulldown of histone H2B. **(a)** GFP pulldown of ARH3 KO cells (two 500 cm^2^ dishes *per* condition) transfected with GFP-ZUD or GFP-zfDi19 and treated with 2 mM H_2_O_2_ (30 min). The elutions (5 min RT) of both pulldowns were immunoblotted for ZUD HRP-coupled, AbD43647 HRP-coupled and ubiquitin antibody. zfDi19 (lacking UIM) shows negligible signal for AbD43647 HRP-coupled, ubiquitin and HRP-ZUD compared to ZUD (zfDi19-UIM), showing the specificity of ZUD for ADP-ribosyl-ubiquitylation. Shown is a representative result from three independent experiments. The GFP input and 95 °C after EDTA elution blots were cropped for clarity. The asterisk (*) indicates the position where the image was cut. Full-length blots are provided in the Source Data of this figure. **(b)** GFP-ZUD pulldown of ARH3 KO cells (eight 500 cm^2^ dishes) transfected with GFP-ZUD and treated with 2 mM H_2_O_2_ (30 min). Immunoblotting the elution with an H2B antibody reveals an NH₂OH-sensitive ADPrUb band. 20% Bis-Tris gel was used to better resolve H2B. Shown is a representative result from three independent experiments. **(a,b)** Following the EDTA elution, GFP-ZUD was eluted by heating the beads at 95 °C for 15 min. Source data

To detect cellular ADP-ribosyl-ubiquitylation by immunoblotting, we engineered a recombinant reagent comprising two ZUD moieties coupled to an HRP-labeled SpyCatcher (Fig. 6a and Extended Data Fig. 6b). Building on our discoveries of ubiquitylation on Ser-ADPr (Figs. 4 and 5) and RNF114 recruitment to DNA lesions^24^, we sought to explore ADP-ribosyl-ubiquitylation in response to DNA damage. Immunoblotting of whole-cell lysates resulted in high background, which hindered the detection of ADP-ribosyl-ubiquitylation (Extended Data Fig. 6c). Encouraged by the improved mono-ADPr detection achieved by combining AbD43647 IgG for immunoprecipitation with its HRP-coupled format^24^, we reasoned that a similar approach—ZUD pulldown followed by immunoblotting with HRP-coupled SpyTag–ZUD—could enable the detection of ADP-ribosyl-ubiquitylation. To investigate the specificity of ZUD, we exploited the chemical stability of Ser-ADPr and the sensitivity of ester bonds to hydroxylamine and boiling^20^ as a means to maintain mono-ADPr conjugation to proteins while releasing ubiquitin from ADP-ribose. Hydroxylamine and boiling completely abolished the ZUD signal. In contrast, these treatments largely preserved the mono-ADPr pattern and the overall ubiquitin signal, while increasing the signal corresponding to short, unconjugated ubiquitin chains (Fig. 6b and Extended Data Fig. 6d). Compared to ZUD, zfDi19 (lacking the UIM) enriches negligible levels of ADP-ribosyl-ubiquitylation, mono-ADPr and ubiquitylation, thereby excluding the possibility that ADP-ribosyl-ubiquitylation is nonspecifically enriched because of strong mono-ADPr binding by the zfDi19 domain in ZUD (Extended Data Fig. 7a). Overall, these experiments demonstrate that ZUD detection following ZUD pulldown is highly specific for ADP-ribosyl-ubiquitylation and ADP-ribose is modified by short ubiquitin chains, primarily dimers and trimers.

To investigate the extent to which the levels of ADP-ribosyl-ubiquitylation depend on DNA damage, we created a bivalent biotin-coupled SpyTag–ZUD reagent (Fig. 6a and Extended Data Fig. 6b), exploiting the strong interaction between biotin and streptavidin for pulldown. Subsequent western blotting with HRP-coupled SpyTag–ZUD allowed us to observe a dramatic increase in ADP-ribosyl-ubiquitylation upon DNA damage (Fig. 6c). Treatment with the PARP1 inhibitor olaparib largely abolished the DNA-damage-induced increase in ADP-ribosyl-ubiquitylation (Fig. 6c), indicating that this composite PTM depends on PARP1 signaling, consistent with our evidence that serine mono-ADPr, produced by PARP1 (refs. ^11,12^), is a target of ester-linked ubiquitylation (Figs. 4 and 5). We observed a similar pattern in ARH3-KO cells with a higher signal for the DNA-damage-treated sample compared to WT cells. As further evidence of its specificity, our ZUD reagent did not detect the main PARP1 band recognized by both anti-PARP1 and mono-ADPr antibodies but rather an upper band corresponding to ADP-ribosyl-ubiquitylated PARP1 (Fig. 6c).

To extend the applicability of our approach beyond PARP1, we greatly upscaled the ZUD pulldown before performing ZUD immunoblotting with hydroxylamine to test specificity. This enabled ZUD detection of ADP-ribosyl-ubiquitylated histones H3 and H2B (Fig. 6d and Extended Data Fig. 7b), for which we identified the modification sites (Fig. 5 and Extended Data Fig. 3c,d).

Overall, we converted ZUD into a versatile reagent for enriching ADP-ribosyl-ubiquitylation, enabling site identification by MS and western blotting detection of this intriguing composite modification.

## Discussion

Recent technological advances have resolved many challenges that have plagued the field of ADPr for decades^6,7^, opening up new research directions within established signaling pathways. This progress is exemplified by the reinterpretation of PARP1 signaling triggered by the discovery of Ser-ADPr by PARP1–HPF1 (refs. ^11–14^) and, more recently, by the concept of mono-ADPr as the second wave of PARP1 signaling^24^. However, investigating the emerging interplay between ADPr and ubiquitylation in cellular contexts requires next-level development of specialized tools and approaches. Only by combining the analysis of the MS behaviors of ADPr using multiple fragmentation techniques with approaches for detecting branched peptides were we able to provide evidence of phosphoribose-linked serine ubiquitylation by *L.* *pneumophila*^11^. Other forms of unconventional ubiquitylation, occurring through labile ester bonds, including the ubiquitylation of ADP-ribose, pose even greater challenges, spanning sample preparation, MS and data analysis. Therefore, it is perhaps not surprising that reports on the exact modification generated by DELTEX enzymes have varied, ranging from conventional lysine ubiquitylation to ADPr of the ubiquitin C terminus, ubiquitylation of the adenine-proximal ribose on various substrates (NAD^+^, free ADP-ribose, ADP-ribosylated peptides and nucleic acids) and direct ubiquitylation of nucleotides without ADP-ribose^30–35^.

Here, we began with our recently developed methods for preserving ester-linked modifications^20^. We introduced the ZUD pulldown, designed a specific EDTA-based elution strategy and developed proteomic approaches tailored to the unique chemistry of this composite modification. This methodology enabled us to identify endogenous ADP-ribosyl-ubiquitylation sites, previously unattainable by MS, even in biochemical reactions. Specifically, we discovered that serine mono-ADPr on histones, PARP1 and other proteins serves as a target for this unconventional PTM, establishing ADP-ribosyl-linked serine ubiquitylation as a cellular signal, including its intriguing role as a dual modification of histones H3 and H2B. The increase in ADP-ribosyl-ubiquitin levels observed in ARH3-KO cells (Fig. 6c), together with in vitro evidence that recombinant ARH3 can remove ADP-ribosyl-ubiquitin from modified peptides^30^, suggests that this Ser-ADPr hydrolase may also act as an eraser of ADP-ribosyl-linked serine ubiquitylation. While the use of ARH3-KO cells has facilitated the identification of ADP-ribosyl-linked serine ubiquitylation, we also found evidence of this composite modification in WT cells, specifically on HMGA1S13 and H2BS6. Intriguingly, toxic levels of ADP-ribosyl-linked serine ubiquitylation may contribute to neurodegeneration in ARH3-deficient patients as a consequence of accumulation of Ser-ADPr^16^ and its readers^24^.

Our identification of ubiquitylation on lysines adjacent to ADP-ribosylated serines (Fig. 2) raises the possibility that high-pH conditions during sample preparation could induce an acyl transfer reaction, leading to the transfer of ubiquitin from the ester bond on ADP-ribose to an isopeptide bond on the adjacent lysine. Given our findings that RNF114 is a reader of ADPr targets, including ADP-ribosyltransferases such as PARP1, PARP12 and tankyrase (Fig. 1), future applications of our methodology are likely to reveal ADP-ribosyl-ubiquitylation sites in other important biological processes, such as interferon signaling. Our discovery of ADP-ribosyl-ubiquitylation sites suggests that RNF114 binders (Fig. 1) are targets of this dual modification. However, some identified proteins may not be direct targets but rather components of complexes containing ADP-ribosyl-ubiquitylated proteins.

Beyond PARP1 and PARP2 signaling, we expect diverse conjugation chemistries, including ADP-ribosyl-ubiquitylation on aspartate and glutamate, as suggested by a recent report that used chemical and enzymatic removal of western blotting signals, albeit without identifying the modification sites^31^. While the original MS evidence of a PTM in cellular contexts represents a key step in fostering new lines of research, as illustrated by the discovery of Ser-ADPr^11,18^, the broad pursuit of such directions relies on the availability of tools that can be readily implemented in any biological laboratory. Complementing sophisticated tools developed by others^6,48^, we previously integrated Ser-ADPr technology with the SpyTag protein ligation system to generate modular, recombinant antibodies^24,25^. In this work, we applied SpyTag technology^46,47^ to engineer ZUD domains as the first specific reagent for detecting this composite PTM by immunoblotting (Fig. 6). Future development of complementary, high-affinity recombinant antibodies through the extension of Ser-ADPr technology could further expand investigations of ADP-ribosyl-ubiquitylation in cellular contexts. We envision that this will ultimately lead to the identification of hundreds of ADP-ribosyl-ubiquitylated targets.

In conclusion, the intricate chemical interplay between ADPr and ubiquitylation is emerging as a promising avenue for both research fields^2^. While the much-studied phosphoribosyl-linked serine ubiquitylation has thus far been exclusively associated with *Legionella* host interactions, our discovery of ADP-ribosyl-linked serine ubiquitylation reveals a signaling mechanism for endogenous processes in mammalian cells. Beyond PARP1 signaling and the DDR, we envision that our multifaceted methodology will uncover this composite PTM in additional biological processes and through diverse attachment chemistries. We propose ADP-ribosyl-ubiquitylation as a general mechanism for cell signaling, with RNF114 acting as its reader in different biological processes.

## Methods

### Cell culture and drug treatments

U2OS cell lines were obtained, authenticated by short tandem repeat profiling and confirmed free of *Mycoplasma* by the American Type Culture Collection cell line authentication services. Cells were routinely tested for *Mycoplasma* contamination. Each cell line was cultured in GlutaMAX DMEM (Thermo Fisher Scientific, 31966021) supplemented with 10% FBS (Gibco) and 100 U per ml penicillin–streptomycin at 37 °C and 5% CO_2_.

To induce DNA damage, cells were incubated with GlutaMAX DMEM supplemented as described above and containing 1–2 mM H_2_O_2_ for 30 min or 60 min. To induce PARP1 inhibition, U2OS WT and ARH3-KO cells were incubated with GlutaMAX DMEM supplemented with 1 µM olaparib (Cayman Chemicals, 10621) for 1 h and 24 h, respectively.

### Plasmids and transfection

The pDEST-CMV-GFP-ZUD plasmid encoding the GFP-tagged zfDi19 and UIM domain of RNF114 (aa 137–228) and the pDEST-CMV-GFP-zfDi19 plasmid encoding GFP-tagged zfDi19 of RNF114 (aa 137–199) for mammalian cell expression was cloned by Gateway cloning.

piTR-RNF114 and piTR-RNF114-C176A encode full-length N-terminal GFP-tagged RNF114 WT and GFP-tagged RNF114-C176A, respectively. Both RNF114 constructs were subcloned from previously established plasmids^24^ into piTR-TTP vector^50^. The piTR-TTP backbone and the transposase encoding pCMV-Trp vector were kindly provided by R. Marschalek (University of Frankfurt).

The pGEX-Spytag3-ZUD plasmid encodes ZUD (aa 138–228 of RNF114). ZUD is N-terminally tagged with GST followed by an HRV3C site, a 6×His tag, Spytag3 and Flag tag. The plasmid was cloned and produced by GeneScript.

### Generation of doxycycline-inducible cells expressing GFP–RNF114 WT and GFP–RNF114-C176A

Previously published RNF114-KO U2OS cells^24^ were complemented with inducible GFP–RNF114 WT or GFP–RNF114-C176A expression using the Sleeping Beauty transposon system^51^. The piTR-RNF114 or piTR-RNF114-C176A plasmids were transfected with a transposase expressing pCMV plasmid in a 10:1 ratio using Lipofectamine 2000 transfection reagent (Invitrogen, 11668019). Then, 24 h after transfection, the cells were selected with 1.5 µg ml^−1^ for 5 days followed by 2 µg ml^−1^ puromycin for 7 days. Then, GFP-negative cells were single-cell-sorted by fluorescence-activated cell sorting into 96-well plates. GFP-negative cells were collected to exclude leaky expression. After sorting, the cells were expanded into six-well plates and tested for inducible expression by adding 1 µg ml^−1^ doxycycline for 24 h to the medium. Cell lysates of each clone either induced or noninduced were prepared using SDS buffer (4% SDS and 50 mM HEPES pH 7.9)^24^ and probed for doxycycline dependent GFP signal by immunoblotting. Clones showing inducible expression upon doxycycline addition were expanded and stored.

### GFP pulldown to identify mono-ADPr-dependent interactors of RNF114

RNF114-KO cell lines^24^ complemented with inducible expression of either GFP–RNF114 WT or GFP–RNF114-C176A were induced using standard medium complemented with 1 µg ml^−1^ doxycycline for 24 h. Afterward, both cell lines were either treated with 1 mM H_2_O_2_ for 60 min or left untreated. For each condition, four biological replicates, each corresponding to a confluent 10-cm dish, were collected and processed in parallel. Cells were collected by washing twice with ice-cold PBS and scraping the cells in 1 ml of ice-cold PBS. The cells were collected and pelleted at 500*g* and 4 °C for 5 min.

Cell lysis was performed in 200 µl of lysis buffer (20 mM HEPES pH 7.9, 300 mM NaCl, 2.5 mM MgCl_2_, 0.5% NP-40, 20% glycerol, 20 µM olaparib, 20 µM ADP-HPD (EMD Millipore, 118415), 2× EDTA-free protease inhibitor cocktail and 750 U per ml Benzonase) by resuspending the cell pellets and incubating them for 1 h on an end-to-end rotator at 4 °C. After lysis, the Benzonase was quenched by adding 200 µl of quenching buffer (20 mM HEPES pH 7.9, 0.5% NP-40, 20 µM olaparib, 20 µM ADP-HPD, 2× EDTA-free protease inhibitor and 30 mM EDTA). The quenched lysate was clarified by centrifuging for 10 min at 20,000*g* and 4 °C. The supernatant was diluted in dilution buffer (20 mM HEPES pH 7.9, 150 mM NaCl, 0.5 mM EDTA, 20 µM olaparib, 20 µM ADP-HPD and 2× EDTA-free protease inhibitor cocktail). Then, 10 µl of ChromoTek GFP-TRAP magnetic particles M-270 (Proteintech, gtd-20) per sample were prewashed three times in 1 ml of washing buffer (10 mM HEPES pH 7.9, 150 mM NaCl and 0.5% NP-40), added to the quenched lysates and incubated on an end-to end rotator for 1 h at 4 °C. After incubation, the supernatant (Flow-Through) was collected and stored at −20 °C. The beads were washed four times in 500 µl of washing buffer and four times in 500 µl of washing buffer without NP-40 to remove residual detergent.

Following resuspension of the beads in 100 µl of digestion buffer (5 ng µl^−1^ trypsin, 50 mM Tris-HCl pH 7.5, 1 mM TCEP and 5 mM CAA), the samples were on-bead digested overnight by incubating each sample at 37 °C and 800 rpm on a shaker.

The supernatant was separated from the beads and used for stage tipping on self-made 30-µg C18 stage tips. The stage tips were activated with 200 µl of methanol, equilibrated twice with 200 µl of elution buffer (30 % acetonitrile in 0.1% formic acid (FA)) and washed twice with 0.1% FA. Afterward, the samples were loaded onto the prepared stage tips and washed twice in 200 µl of 0.1% FA. Peptides were eluted in 100 µl of elution buffer and fully dried in a vacuum concentrator (Eppendorf).

One fifth of each sample was injected into a Q-Exactive HF (Thermo Fisher) for MS/MS measurement. The setup of liquid chromatography (LC) for MS is explained later. The peptides were separated by reverse-phase LC using a 26-min gradient from 4% buffer B to 30% buffer B in 20 min followed by a high-organic wash phase. DIA data were collected in positive mode with a resolution of 15,000, an automatic gain control (AGC) target of 3 × 10^6^ and a maximum injection time of 22 ms. Method details are also stored in the raw files provided.

The resulting raw data were analyzed with DIA-NN^52^ using the following settings: UP000005640_9606.fasta of the human proteome was used for the generation of a library with the ‘reannotation’ option enabled. Options for ‘FASTA digest for library free search/library generation’ and ‘deep learning-based spectra, RTs and IM prediction’ were enabled. Trypsin/P was set as a protease and two missed cleavages of the tryptic digest were allowed, along with one variable modification (oxidation of methionine or acetylation of the N terminus). Further ‘N-term M excision’ and ‘C carbamidomethylation’ were set as fixed modifications. Peptides between 5 and 30 aa, precursors carrying a charge of 1–4 in a range of 300–800 *m*/*z* and fragment ions in a range of 200–1,800 *m*/*z* were considered for the analysis. Mass accuracy and MS1 accuracy were set to default values. The options ‘use isotopologs’, ‘match-between-runs’, ‘heuristic protein interference’ and ‘no shared spectra’ were enabled. Protein inference was set to ‘genes’, neural network classifier was set to ‘single-pass mode’, quantification strategy was set to ‘robust LC (high precision)’, cross-run normalization was set to ‘RT-dependent’, library generation was set to ‘smart profiling’ and speed and RAM usage were set to ‘optimal results’.

The protein group output of DIA-NN was used for analysis of the interactors using a custom R script and RStudio. Briefly, limma^53^ was used for significance testing of log_2_ intensities and the adjusted *P* values corrected for multiple testing were filtered for significance if adjusted *P* < 0.05 was fulfilled (corresponding to −log_10_(adjusted *P* value) > 1.3).

### Open search of published datasets

An open search for potential combinations of ADPr and ubiquitin in published datasets was performed using MSFragger (version 22.0)^54^. For the presented open search, the previously published dataset PXD023835 (ref. ^38^) was used. All the corresponding raw files were analyzed using the following settings with the Database and MSFragger modules of Fragpipe^55^. As the database, we used an automatically downloaded FASTA of the human proteome (UP000005640) including contaminants and decoys added by Fragpipe.

For ‘peak matching’, the following parameters were set: precursor mass tolerance from −1 to 1,000 Da, fragment mass tolerance of 20 ppm and isotope errors set to 0/1/2.

For ‘protein digestion’, the following parameters were set: trypsin as protease allowing two missed cleavages and a peptide length of 7–50 with a mass range of 500–5,000 Da.

For modifications, the maximum number of variable modifications was set to three with oxidation of methionine as a variable modification and the maximum combinations parameter was kept on default settings. Carbamidomethylation of cysteine residues was set as fixed modification.

For ‘open search options’, the following parameters were set: ‘report mass shift as a variable mod’ was turned off, ‘track zero top *N*’ and ‘zero bin accept expect’ were set to 0, ‘zero bin multiply expect’ was set to 1 and ‘delta mass exclude range’ was set to −1.5–1.5. ‘Localize mass shift’ was turned on.

For ‘advanced peak matching’, the following parameters were set: ‘min frags modeling’ was set to 2, ‘deisotope’ was enabled, ‘deneutrallos’ remained on default, ‘minimum matched frags’ was set to 7 and the ‘max fragment charge’ was set to 4. Fragment ion series allowed were *c*, *z*, *b* and *y*. No custom ion series was added. ‘Precursor true tolerance’ remained at 20 ppm. ‘Override charge with precursor charge’ was disabled.

All the output files were merged into one Excel table and filtered to remove reverse hits marked with rev_sp. Then, the mass shift column was filtered for 541 as a proxy for ADPr, 655.1 as a proxy for combinations of ADPr and GlyGly and 924.2 as a proxy for combinations of ADPr and LRGG. Duplicate peptides were removed to determine the number of unique peptides identified for each of the three mass shifts.

Subsequently, a MaxQuant^43^ search was performed to validate the findings by localizing the modifications and gaining annotated MS2 spectra. The same dataset was analyzed with MaxQuant. Every setting was kept on default unless stated differently. As variable modifications, ADPr was allowed on serine residues and ubiquitylation GlyGly and LRGG were allowed on lysine residues. Additionally, oxidation of methionine and acetylation of the protein N terminus were considered as variable modifications. Carbamidomethylation of cysteine residues was set as a fixed modification. The maximum number of modifications per peptide was set to three. Trypsin was set as the protease and five missed cleavages were allowed. We used a FASTA database for the human proteome from UniProt (UP000005640_9606). The maximum peptide length was set to five and the maximum peptide mass to 8,000 Da. The ‘second peptides’ option was enabled for identification.

### Coupling of Spy-tagged ZUD with HRP-tagged and biotin-tagged SpyCatcher

To couple Spy-tagged ZUD with either HRP–SpyCatcher (Bio-Rad, TZC002) or biotin–SpyCatcher (Bio-Rad, TZC002), the purified Spy-tagged ZUD was diluted to to 1 µg µl^−1^ in 1× PBS. Then, the desired SpyCatcher was added at a 1:10 (v/v) ratio to the diluted recombinant protein; for example, 10 µl of SpyCatcher was added to 100 µl of Spy-tagged ZUD. The solution was incubated at room temperature for 1 h, aliquoted and stored at −20 °C until further use.

### GFP pulldown for site identification with urea elution

WT U2OS cells equivalent to two confluent 15-cm dishes were transfected using PEI with 25 µg of plasmid per dish encoding GFP-tagged ZUD (1:3 DNA:PEI ratio in OptiMEM, Gibco) and collected as described for the RNF114 interactome. To preserve ester-linked PTMs, we used our previously described conditions^20^. Until the elution, every step was performed as described above (GFP pulldown to identify mono-ADPr-dependent interactors of RNF114). Elution was then performed by resuspending the beads in 25 µl of urea elution buffer (8 M urea, 20 mM HEPES pH 7.0 and 1 mM DTT) for 10 min at 37 °C. Afterward, the supernatant was separated from the beads and diluted tenfold using digestion buffer optimized for ArgC digestion (ammonium acetate pH 5.0 and 5 mM DTT). The elution was digested by adding 0.25 µg of ArgC and 1 µg of LysC for 3 h at 37 °C and stopped by adding FA to a final concentration of 2%. Stage tipping, drying and resuspension of the peptides were performed as described for the interactor screen. Resuspended peptides were injected into a Q-Exactive HF Oribitrap MS instrument for data-dependent acquisition (DDA). The machine was operated in positive mode. For reverse-phase LC, a 153-min gradient of increasing buffer B was used, which increased to 50% in 120 min followed by an increase to 90% over 30 min and a 3-min wash with 90% buffer B. Data were collected along the gradient of 0–128 min. The scan range for full MS spectra was 400–1,600 m/z with an AGC target of 100,000 and a maximum injection time of 100 ms. For MS2 scans, a Top*N* (*N* = 5) method was chosen using a scan range of 200–2,000 *m*/*z* with a fixed first mass of 120 *m*/*z* to ensure measurement of adenine peaks. Method details are also stored in the raw files provided.

### Optimized GFP pulldown and EDTA elution

ARH3-KO U2OS cells were transfected with plasmids encoding GFP–ZUD or GFP–zfDi19 using PEI and collected as described above. Instead of transfection, RNF114 KO complemented with inducible GFP–RNF114 expression was induced as described above. DNA damage treatment was performed with 2 mM H_2_O_2_ for 30 min. For each pulldown, cells were grown confluent on 500-cm^2^ square dishes. The numbers of used dishes are stated in the respective figure legends. The following protocol describes the processing of one 500-cm^2^ dish and volumes can be scaled up and down accordingly. Cell pellets were resuspended in 1 ml of lysis buffer (20 mM HEPES pH 7.0, 300 mM NaCl, 2.5 mM MgCl_2_, 0.5% NP-40, 20% glycerol, 750 U per ml benzonase, 2 µM olaparib, 2 µM ADP-HPD, 50 µM PR-619 (Sigma-Aldrich, SML0430-1MG) and 2× EDTA-free protease inhibitor cocktail) and incubated on an end-to-end rotator for 1 h at 4 °C. Afterward, the lysate was diluted by adding 1 ml of dilution buffer (20 mM HEPES pH 7.0, 0.5% NP-40, 2 µM olaparib, 2 µM ADP-HPD, 50 µM PR-619 and 2× EDTA-free protease inhibitor cocktail). The diluted lysates were clarified by centrifugation at 16,000*g* for 5 min at 4 °C, followed by collection of the supernatant. Next, 100 µl of ChromoTek GFP-TRAP magnetic particles M-270 per square dish were incubated with the diluted lysate for 1 h at 4 °C on an end-to-end rotator. After incubation, the beads were washed four times in ice-cold wash buffer (20 mM HEPES pH 7.0, 300 mM NaCl and 0.05% NP-40) followed by three washes in ice-cold wash buffer without NP-40 (20 mM HEPES pH 7.0 and 300 mM NaCl). One last wash was performed with wash buffer without NP-40 at room temperature for 15 min and 900 rpm on a shaker. Proteins were eluted by incubating the beads with 100 µl of EDTA elution buffer (30 mM EDTA, 20 mM HEPES pH 7.0 and 300 mM NaCl) for 5 min at room temperature at 900 rpm on a shaker. Note that multiple elution strategies were tested; differing elution times and temperatures are stated in the figure legends. For proteomics, the eluted proteins were alkylated by adding 100 µl of digestion buffer (4 M urea, 20 mM ammonium acetate, 5 mM DTT and 15 mM CAA) and incubated in the dark for 1 h at room temperature. Excess CAA was quenched by adding DTT to a final concentration of 15 mM and incubation in the dark for 10 min at room temperature. The alkylated elutions were digested for 3 h at 37 °C by adding either only 0.3 µg of ArgC or 0.3 µg of ArgC and 1.2 µg of LysC per 500-cm^2^ dish. Unless otherwise stated, the digest was performed with ArgC and LysC. Digestions were stopped by adding FA to a final concentration of 2% and stored at −20 °C until stage tipping. Stage tipping and dyring of the peptides were performed as described above.

### Pulldown with biotin-coupled ZUD reagent

To pull down endogenous proteins carrying ADP-ribosyl-ubiquitylation, 25 µg of biotin-coupled ZUD reagent per 15-cm dish was used. Two confluent 15-cm dishes per condition of either ARH3-KO U2OS or WT U2OS cells were used. ARH3-KO U2OS and WT U2OS cells were treated with olaparib and 2 mM H_2_O_2_ for 30 min and collected as described. The pulldown followed the optimized pulldown protocol. However, the biotin-coupled ZUD reagent was incubated during lysis with streptavidin magnetic beads (1:1, biotin-coupled ZUD reagent and Streptavidin beads (w/v); Invitrogen Dynabeads MyOne Streptavidin C1, 65-002). After preparing the beads, they were washed three times in wash buffer with NP-40 to remove unbound biotin–ZUD from the beads that could compete with the bead-coupled biotin–ZUD to bind endogenous proteins carrying ADP-ribosyl-ubiquitylation. The beads were then equally distributed to the samples and the protocol of optimized GFP pulldown and EDTA elution was performed.

### Immunoblotting

For immunoblotting, EDTA elutions were supplemented with 4× LDS loading buffer (NuPAGE LDS sample buffer, Invitrogen, NP0007) with 100 mM DTT to a final concentration of 1× LDS loading buffer and 25 mM DTT. Before gel loading, the samples were incubated at room temperature for 5–15 min. Where stated, the EDTA elution was split in two. One half was heated (5 min at 95 °C on a shaker at 900 rpm) before loading. Where stated, one half of the elution was treated with 1 M hydroxylamine (2 h at room temperature on a shaker at 900 rpm). The same volume of water was added to the other half of the elution.

SDS–PAGE and immunoblotting were performed in precooled buffers on ice. mPAGE 4–12% Bis–Tris precast gels (Millipore, MP41G15) were used. The 20% and 8% Bis–Tris gels were self-casted. After SDS–PAGE, the gels were transferred by wet transfer at 110 V for 90 min onto nitrocellulose membranes (Amersham, 10600001) or, in the case of the VU-1 antibody, PVDF membranes (Amersham, 10600030) were used according to the manufacturer’s instructions. PVDF membranes were treated with 0.5% glutaraldehyde in PBS for 20 min before blocking. Membranes were blocked with 5% milk in PBS-T (PBS and 0.1% Tween-20) for 1 h at room temperature and incubated at 4 °C overnight with the respective primary antibodies. If required, the membranes were incubated with secondary antibodies for 1 h at room temperature.

The following primary antibodies and detection reagents were used for immunoblotting: mouse anit-GFP Living Colors monoclonal antibody (JL-8) (Takara Bio, 632381; 1:3,000), rabbit anti-PARP1 (Abcam, ab6079; 1:1,000), HRP-coupled anti-mono-ADP-ribose (Bio-Rad, AbD43647 (ref. ^24^); 0.03 µg ml^−1^), rabbit anti-ADPRHL2 (ARH3) (Merck, HPA027141; 1:1,000), HRP-coupled ZUD reagent (1 µg ml^−1^), rabbit anti-histone H3 (Cell Signaling Technology, 9715; 1:1,000), rabbit anti-histone H2B (D2H6) (Cell Signaling Technology, 12364; 1:1,000), HRP-coupled anti-histone H3 (D1H2) (Cell Signaling Technology, 12648; 1:1,000), Anti-ubiquitin antibody clone VU-1 (Life Sensors, VU101; 1 µg ml^−1^).

The following secondary antibodies were used: Amersham enhanced chemiluminescence (ECL) mouse IgG HRP-linked whole antibody (Amersham, NA931-1ML; 1:8,000) and Amersham ECL rabbit IgG HRP-linked whole antibody (Amersham, NA934-1ML; 1:8,000).

All antibody and detection reagents were diluted in 5% milk in PBS-T.

### LC–MS systems

Orbitrap Lumos and Oribtrap Q-Exactive HF MS instruments were connected to an Easy-nLC 1200 (Thermo Scientific) chromatographic system. The Orbitrap Fuison was connected to an Easy-nLC 1000 chromatographic system (Thermo Scientific). Columns (Poroshell 120-EC C18 medium, 2.7-μm particle diameter) were packed in CoAnn 75-μm fritless emitters (MSWil, TIP36007515-20-5) and had a length of 35 cm (Orbitrap Lumos) or 20 cm (Orbitrap Q-Exactive HF and Oribtrap Fusion). The running buffers were 0.1% FA (buffer A) in water and 0.1% FA and 80% acetonitrile (Buffer B) in water. All data were collected in positive mode.

### Multistage triggered MS methods to detect ADPr-ribosyl-ubiquitylation

To focus data acquisition time on the peptides of interest, we performed a multistage methodology. Methods aimed to trigger specifically on peptides carrying ADP-ribosyl-ubiquitylation were set up for HCD and ETD in DDA mode on an Oribtrap Fusion Lumos MS instrument (Thermo Scientific) equipped with a FAIMS Pro Duo interface (Thermo Scientific). The compensation voltages of the FAIMS were set to −50 V and −70 V.

The following gradient was used for the triggered methods: 1–31% buffer B increase over 90 min followed by an increase in buffer B to 50% over 10 min and another 10-min increase to 95% buffer B, finalized with a 20-min wash with 95% buffer B. For ArgC digests, the gradient was adjusted to account for the presence of longer and more hydrophobic peptides: 1–40% buffer B increase over 90 min followed by an increase in buffer B to 50% over 10 min and another 10-min increase to 95% buffer B, which was followed by a 20-min wash with 95% buffer B. Method details are also stored in the raw files provided.

### Adenine-triggered HCD spectra

MS1 spectra from 350–1,550 *m*/*z* were collected with an AGC target of 100,000 and a maximum injection time of 50 ms. Precursors with charge states of 2–7 were rapidly fragmented by HCD (injection time of 22 ms and an AGC target of 50,000) in a Top20 method. The MS2 scan range started at 120 *m*/*z* to detect the adenine diagnostic ion, if present. If a mass of 136.062 (Adenine) with a mass tolerance of 15 ppm was detected in this initial MS2 scan, a second HCD fragmentation was triggered on the same precursor. For the second MS2 scan, the maximum injection time was set 300 ms and an AGC target of 150,000 to increase the quality of adenine-containing precursors.

### High-quality triggered HCD and ETD spectra for ADP-ribosyl-ubiquitylated peptides

Further focus was achieved with another layer of trigger specificity for higher-quality HCD and ETD spectra. Data were collected in a fashion similar to that described above, with triggered ETD methods limited to precursors with a charge of 3 and higher. Rapid MS2 scans were again collected at Top20. Upon the detection of 136.062 (adenine), a second HCD fragmentation was triggered on the same precursor with the maximum injection time set to 200 ms and the AGC target set to 150,000. If this second MS2 scan yielded *m*/*z* peaks of either 364.134 (AMP-GlyGly) or 462.11 (ADP-GlyGly), an additional MS2 scan using either ETD or HCD was performed on this precursor with a maximum injection time of 300 ms and an AGC target of 150,000.

### Alkylation assay with H3_(22–44)_ S28-ADPr peptide

First, 1 µg of a biotinylated ADP-ribosylated H3 peptide 22–44 (Anaspec, AS-64440-1), prepared as previously described^24,25^, was incubated in 4 M urea, 20 mM ammonium acetate, 5 mM DTT and 15 mM CAA for 1 h in the dark at room temperature. Excess CAA was quenched by adding DTT to a final concentration of 15 mM and incubating for 10 min in the dark at room temperature. In parallel, the same experiment was performed without CAA. The peptides were stage-tipped and dried as described above and used for MS.

### MaxQuant analysis to detect ADP-ribosyl-ubiquitylation

To detected ADP-ribosyl-ubiquitylation, MaxQuant^43^ version 2.4.12 was used. A modification was set up integrating our observations on the fragmentation behavior of ADP-ribosyl-ubiquitylation. The composition of ADP-ribosyl-ubiquitylation was set to H_27_O_15_P_2_C_19_N_7_, resulting in a neutral mass of 655.104 Da. Neutral losses and diagnostic ions were set for the whole modification (M + H + 656.111 Da), ADP-GlyGly (H_21_N_7_O_12_P_2_C_14_, 541.072 Da; M + H + 542.079 Da), AMP-GG (H_20_N_7_O_9_PC_14_, 461.106 Da; M + H + 462.113 Da) and adenosine-GlyGly (H_17_N_7_O_5_C_14_, 363.129 Da; M + H + 364.136 Da). Diagnostic ions were also set up for adenine (H_5_N_5_C_5_, 135.054 Da; M + H + 136.061), loss of GlyGly + H_2_O (H_8_O_3_N_2_C_4_, 132.053 Da; M + H + 133.060 Da) and the corresponding adenosine-H_2_O (H_10_C_10_O_2_N_5_, 232.083 Da; M + H + 233.090), which was observed in MS2 spectra, and conventional adenosine (H_12_C_10_O_3_N_5_, 250.094 Da; M + H + 251.101). Moreover, neutral losses and diagnostic ions for a potential form of adenosine with a loss of the GlyGly remnant (H_11_C_10_O_3_N_5_, 249.086 Da; M + H + 250.093) was set up. ADPr-ribosyl-ubiquitylation was allowed on serine residues. To perform the MaxQuant search, the described modification was used as a variable modification alone or in combination with, for example, monomethylation and dimethylation or acetylation, which are predefined in the software. We allowed up to five variable modifications per search. The library digestion was adjusted according to the digestion method with either ArgC or ArgC and LysC, allowing up to eight missed cleavages and a peptide range of 5–30 aa with a maximum mass of 8,000 Da. As a protein sequence database, the human proteome (UP000005640_9606) from UniProt was used. To boost identification rates, match-between runs and second peptide search were switched on.

### Purification of Spy-tagged ZUD

BL21 *Escherichia coli* cells were transformed with 1 µg of pGEX-Spytag3-ZUD plasmid. For purification of GST-tagged and Spy-tagged ZUD, a previously described purification protocol was used^24^. After elution, the buffer of the purified protein was exchanged to wash buffer (50 mM HEPES pH 7.9, 200 mM NaCl and 1 mM DTT) in an Amicon ultracentrifuge filter (30-kDa molecular weight cutoff; Millipore, UFC5030) by centrifuging three times at 7,000*g* for 5 min and 4 °C. For the second and third centrifugation step, 300 µl of wash buffer was added to the filter. After the last filtering step, roughly 100 µl of supernatant was left on the filter, containing purified ZUD. The supernatant was aliquoted, frozen using dry ice and stored at −80 °C.

### In vitro reactions to modify H3 peptides and nucleosomes

ADP-ribosyl-ubiquitylation peptides and nucleosomes were produced as previously described^24,25^. For generation of ADP-ribosyl-ubiquitylation on peptides, the biotinylated ADP-ribosylated H3 peptide 1–21 (Anaspec, AS-64611-1) was used. Previously generated nucleosomes^24^ containing ADP-ribosylated H3 on S10 were subjected to an ADP-ribosyl-ubiquitylation reaction^30^. Then, 0.25 µM UBE1 (Boston Biochem, E-305-025), 1.25 µM UBCH5A (Boston Biochem, E2-616-100), 50 µM ubiquitin (Scientific Laboratory Supplies, U5507-1MG), 2.5 µM DTX2 RING-DTC and 2 µg of biotinylated H3_(1–21)_S10ADPr peptide were incubated at 37 °C for 30 min in 50 mM HEPES pH 7.2, 50 mM NaCl, 5 mM MgCl_2_ and 1 mM DTT, with or without 1 mM ATP, as indicated. For the H3S10ADPr nucleosome, the same concentrations were used except for 2 µg of ubiquitin, 0.5 µM DTX3L RING-DTC and 1 µg of H3S10ADPr nucleosome. Recombinant DTX2 RING and DTX3L RING-DTC were kindly provided by I. Ahel (University of Oxford). The H3S10 peptide reaction was digested with ArgC, while the nucleosome reaction was digested with ArgC and LysC. Both digests were followed by stage tipping as described for the optimized GFP pulldown. LC–MS/MS was performed with a Q-Exactive HF (Thermo Fisher) instrument for HCD fragmentation and an Orbitrap Fusion (Thermo Fisher) instrument for ETD fragmentation. The following 53-min gradient was used for LC–MS/MS with the Q-Exactive HF: increase in buffer B to 40% in 40 min, followed by a 5-min increase in buffer B to 55%, a subsequent 5-min increase to 90% buffer B and an additional 3-min wash with 90% buffer B. A Top5 method was used for DDA measurements. For MS2 scans, the AGC target was set to 1,000,000 and the maximum injection time was set to 200 ms. The first fixed mass was 120.0 *m*/*z* to include adenine peaks. The following 50-min gradient was used for LC–MS/MS with the Orbitrap Fusion: increase in buffer B to 50% in 40 min, followed by a 5-min increase in buffer B to 100% and an additional 5-min wash with 100% buffer B. To focus the analyses on peptides carrying ADP-ribosyl-ubiquitylation, precursor ions with *m*/*z* of 476.848 or 495.532 were selected for MS2 scans. For MS2 scans, the AGC target was set to 100,000 and the maximum injection time was set to 300 ms. The first fixed mass was 120.0 *m*/*z*. Exact method details are also stored in the raw files provided. The raw files were analyzed with MaxQuant using a FASTA file containing the proteins used for the *in vitro* reaction and the H3S10 peptide sequence ‘ARTKQTARKSTGGAPRKQLAGGK’.

### Data analysis

Data analysis was performed using RStudio, Prism 9, Excel and Image Lab.

### Reporting summary

Further information on research design is available in the Nature Portfolio Reporting Summary linked to this article.

## Online content

Any methods, additional references, Nature Portfolio reporting summaries, source data, extended data, supplementary information, acknowledgements, peer review information; details of author contributions and competing interests; and statements of data and code availability are available at 10.1038/s41589-025-01974-5.

## Supplementary information

Supplementary Information Supplementary Figs. 1–4.

Reporting Summary

Supplementary Data 1 Proteomics data.

## Source data

Source Data Fig. 3 Unprocessed western blots.

Source Data Fig. 6 Unprocessed western blots.

Source Data Extended Data Fig. 1 Unprocessed western blots.

Source Data Extended Data Fig. 2 Unprocessed western blots.

Source Data Extended Data Fig. 6 Unprocessed western blots.

Source Data Extended Data Fig. 7 Unprocessed western blots.

## Data Availability

The MS proteomics data were deposited to the ProteomeXchange Consortium through the PRIDE^56^ partner repository with the dataset identifier PXD058858. Source data are provided with this paper.

## Code availability

Code used in this manuscript was deposited to the ProteomeXchange Consortium through the PRIDE^56^ partner repository with the dataset identifier PXD058858.
