# Publisher Correction: Serine ADPr on histones and PARP1 is a cellular target of ester-linked ubiquitylation

**DOI:** 10.1038/s41589-025-02024-w

**Published:** 2025-08-21

**Authors:** Andreas Kolvenbach, Maria Dilia Palumbieri, Thomas Colby, Diyaraj Nadarajan, Remo Bode, Ivan Matić

**Affiliations:** 1 Max Planck Institute for Biology of Ageing, Cologne, Germany; 2 Cologne Excellence Cluster for Aging and Aging-Associated Diseases (CECAD), University of Cologne, Cologne, Germany

**Keywords:** Proteomics, Post-translational modifications, Cell signalling, Mass spectrometry, Histone post-translational modifications

Correction to: *Nature Chemical Biology* 10.1038/s41589-025-01974-5, published online 9 July 2025.

In the version of this article initially published, the α configurations shown in Fig. 2a, c, d, Fig. 4b, c and Fig. 5a, b were incorrectly presented in a β orientation and are now updated in the HTML and PDF versions of the article.

