# Supplementary material for: Serine ADPr on histones and PARP1 is a cellular target of ester-linked ubiquitylation
Source: Nat Chem Biol. 2025 Jul 9;21(11):1762–72. doi: 10.1038/s41589-025-01974-5 (PMC12568645; doi:10.1038/s41589-025-01974-5)
Supplement: Supplementary file 1 — Supplementary Figs. 1–4. [file 41589_2025_1974_MOESM1_ESM.pdf]

nature chemical biology

Supplementary information https://doi.org/10.1038/s41589-025-01974-5

Serine ADPr on histones and PARP1 is a cellular target of ester-linked ubiquitylation

In the format provided by the authors and unedited

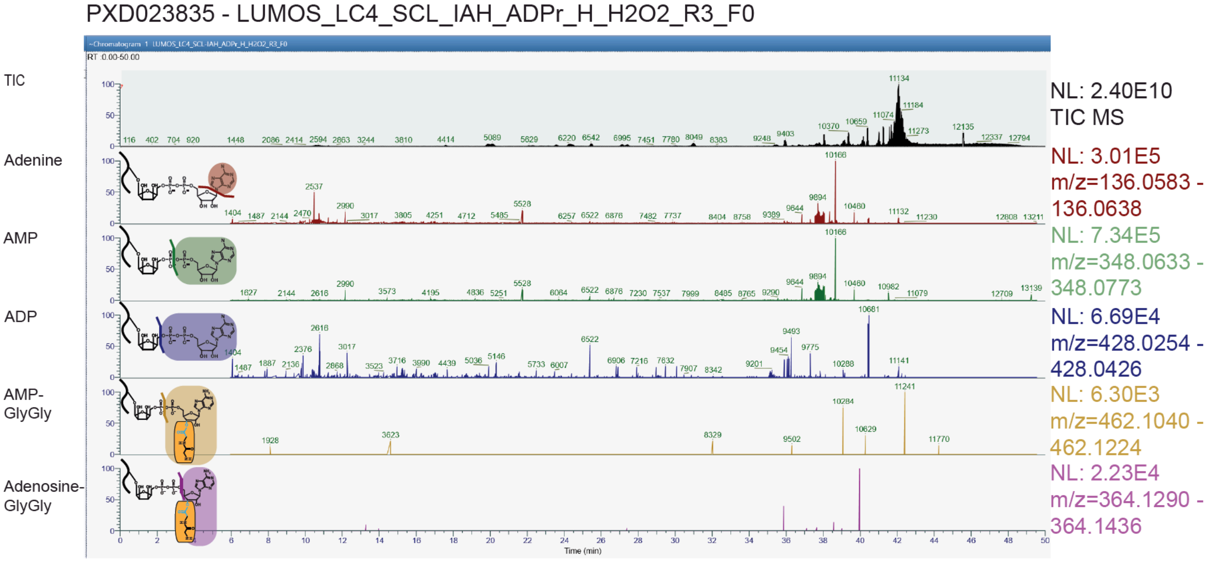

Supplementary Figure 1: Representative Freestyle layout showing the abundance of conventional ADPr diagnostic ions (Adenine, AMP, ADP) and ADP-ribosyl-ubiquitylation (Adenine, AMP-GlyGly, Adenosine-GlyGly) diagnostic ions occurring in a MS/MS analysis of one sample of the published dataset PXD023835. The highlighted parts of mono-ADPr or ADP-ribosyl-ubiquitylation depict the parts of both modifications that result in diagnostic ions.

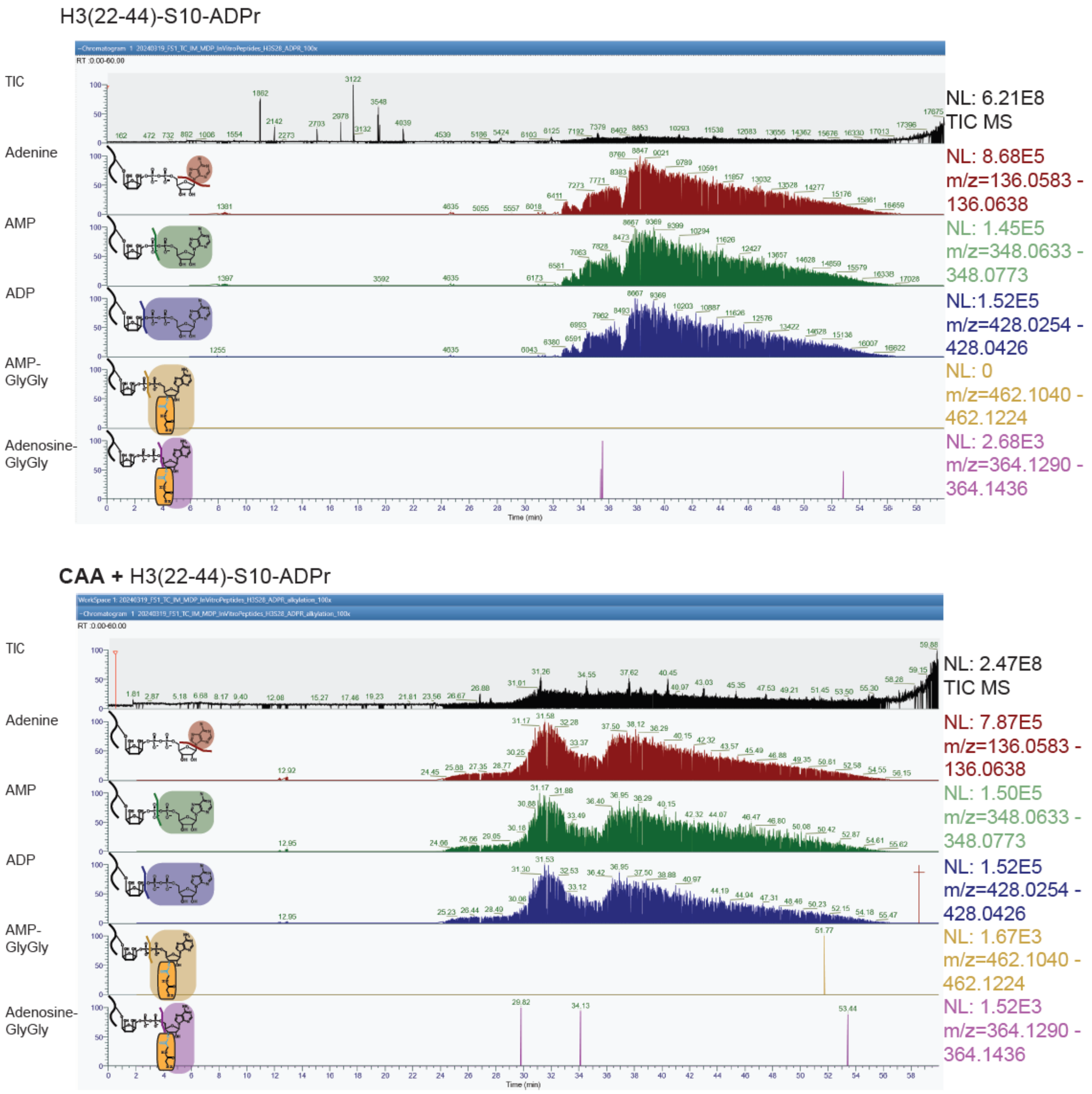

Supplementary Figure 2: Alkylation of ADP-ribosylated H3S10 peptides with CAA did not induce the presence of artifact ions with m/z values corresponding to ADP-ribosyl-ubiquitylation diagnostic ions as assessed by using a Freestyle Layout.

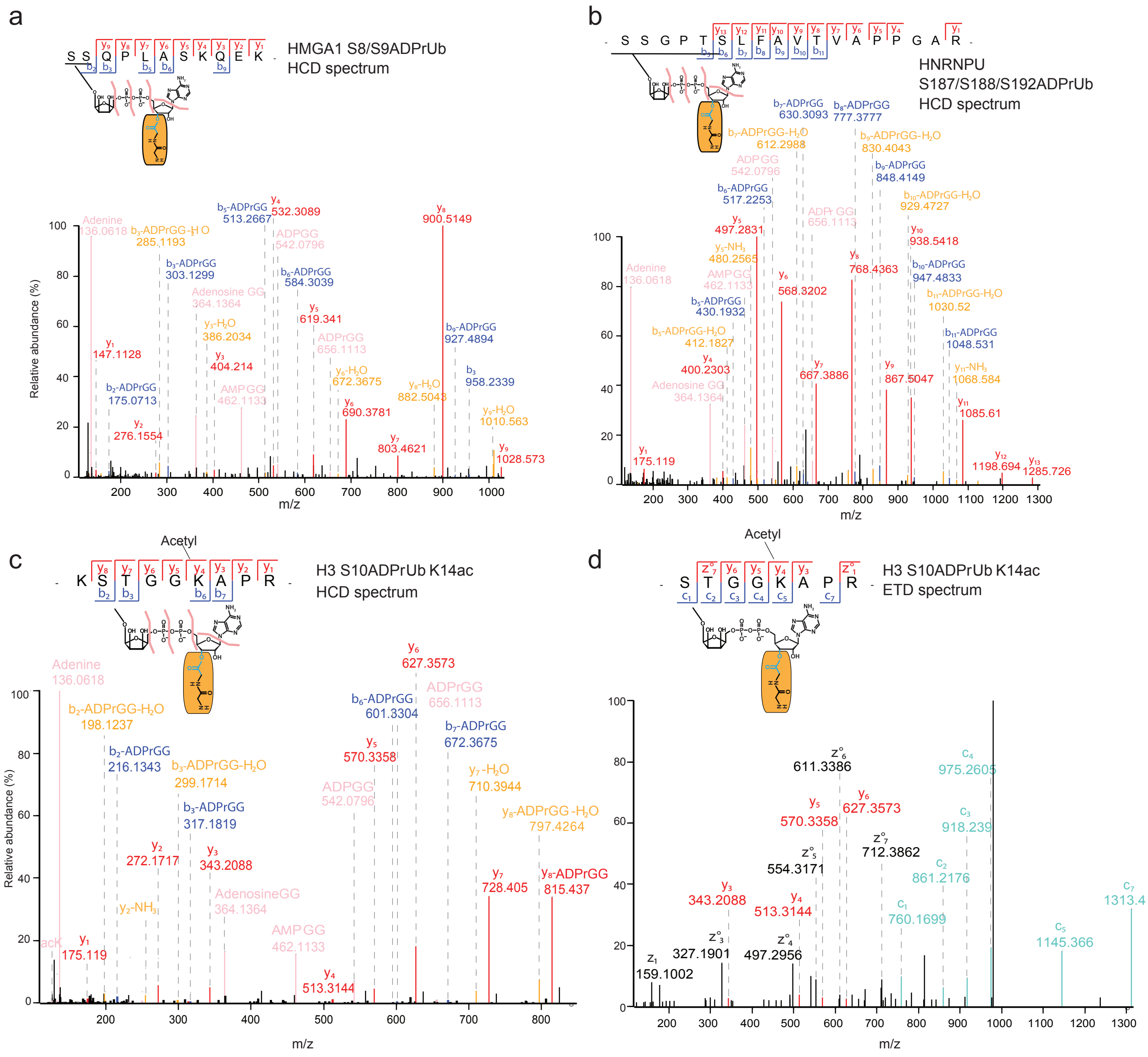

Supplementary Figure 3: Additional spectra of peptides modified with ADP-ribosyl-ubiquitylation. **(a,b)** HCD spectra of ADP-ribosyl-ubiquitylation likely on serine 8 or 9 of HMGA1 and of serine 187, 188 or 192 on HNRNPU. All shown HCD spectra contain the diagnostic ions of ADP-ribosyl-ubiquitylation. The ETD spectra do not contain diagnostic ions as the modification stays intact and is not fragmented, thus allowing the localization of the modification site. These spectra illustrate that ETD data is critical to localize the ADP-ribosyl-ubiquitylation on peptides. However, the presence of ADP-ribosyl-ubiquitylation can be validated by diagnostic ions using HCD. This illustrates how HCD and ETD data can complement each other. **(a,b)** EDTA elution: 15 min at 37 °C. **(a)** One 500 cm^2 dish of doxycycline-induced GFP-RNF114 WT cells, was treated with 2 mM H_2O_2 treated (30 min). **(b)** two 500 cm^2 dishes of ARH3 KO cells transfected with GFP-ZUD were treated with 2mM H_2O_2 (30 min).

(c,d) HCD and ETD spectra of ADP-ribosyl-ubiquitylation on serine 10 of H3 occurring in close proximity to acetylation of lysine 14, both modifications are confidently localized by ETD. **(c)** EDTA elution: 20 min at RT and ArgC digested. ARH3 KO cells (three 500 cm^2 dishes) transfected with GFP-ZUD were treated with 2mM H_2O_2 (30 min). **(d)** EDTA elution: 15 min at 37 °C. ARH3 KO cells (two 500 cm^2 dishes) transfected with GFP-ZUD were treated with 2mM H_2O_2 (30 min).

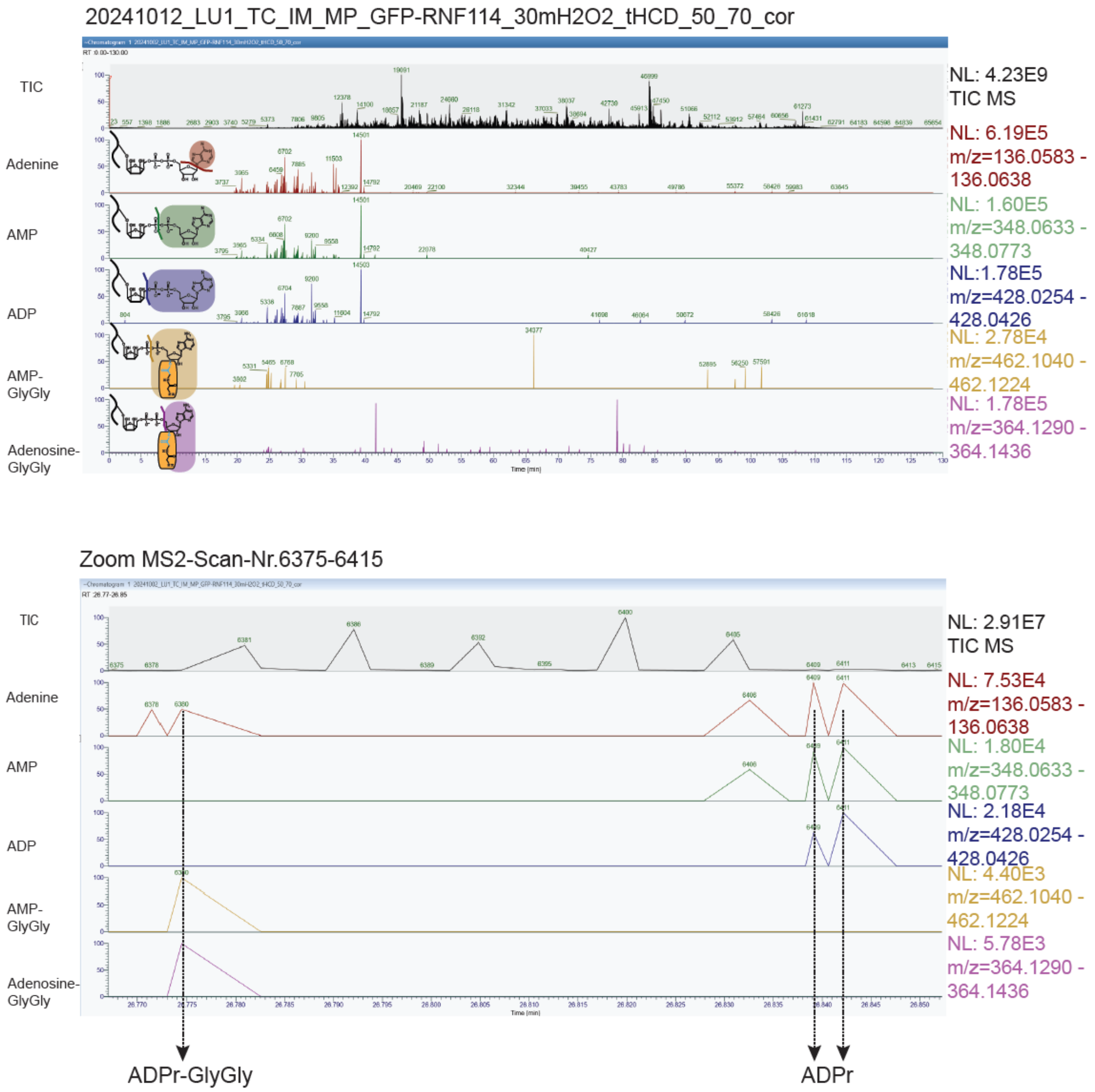

Supplementary Figure 4: The presence of diagnostic ions originating from ADPr-GlyGly or mono-ADPr can be quickly validated and distinguished by inspecting the raw data with Freestyle, using a layout that highlights the respective m/z peaks observed in MS2 spectra. This is particularly useful if peptides carry additional modifications, such as di-methylation, as seen in the case of H3.
