# Supplementary material for: Serine ADPr on histones and PARP1 is a cellular target of ester-linked ubiquitylation
Source: Nat Chem Biol. 2025 Jul 9;21(11):1762–72. doi: 10.1038/s41589-025-01974-5 (PMC12568645; doi:10.1038/s41589-025-01974-5)
Supplement: Supplementary file 2 — Reporting Summary [file 41589_2025_1974_MOESM2_ESM.pdf]

nature portfolio

Corresponding author(s): Ivan Matić

Last updated by author(s): Jun 6, 2025

Reporting Summary

Nature Portfolio wishes to improve the reproducibility of the work that we publish. This form provides structure for consistency and transparency in reporting. For further information on Nature Portfolio policies, see our Editorial Policies and the Editorial Policy Checklist.

Statistics

For all statistical analyses, confirm that the following items are present in the figure legend, table legend, main text, or Methods section.

n/a	Confirmed	
☐	☒	The exact sample size (n) for each experimental group/condition, given as a discrete number and unit of measurement
☐	☒	A statement on whether measurements were taken from distinct samples or whether the same sample was measured repeatedly
☒	☐	The statistical test(s) used AND whether they are one- or two-sided<br>*Only common tests should be described solely by name; describe more complex techniques in the Methods section.*
☒	☐	A description of all covariates tested
☒	☐	A description of any assumptions or corrections, such as tests of normality and adjustment for multiple comparisons
☐	☒	A full description of the statistical parameters including central tendency (e.g. means) or other basic estimates (e.g. regression coefficient) AND variation (e.g. standard deviation) or associated estimates of uncertainty (e.g. confidence intervals)
☐	☒	For null hypothesis testing, the test statistic (e.g. F, t, r) with confidence intervals, effect sizes, degrees of freedom and P value noted<br>*Give P values as exact values whenever suitable.*
☒	☐	For Bayesian analysis, information on the choice of priors and Markov chain Monte Carlo settings
☒	☐	For hierarchical and complex designs, identification of the appropriate level for tests and full reporting of outcomes
☒	☐	Estimates of effect sizes (e.g. Cohen's d, Pearson's r), indicating how they were calculated

Our web collection on statistics for biologists contains articles on many of the points above.

Software and code

Policy information about availability of computer code

Data collection	No custom software was used, all software used in this manuscript is publicly available and described in the methods. Software used: data collection for mass-spectrometry was performed with Thermo Scientific Xcalibur, data collection for immunoblotting was performed with Image Lab.
Data analysis	No custom software was used, all software used in this manuscript is publicly available and described in the methods. Software used: GraphPad Prism (version 9), ImageJ (version 2.14.0/1.54f), Microsoft Excel (version 16.97.2), Image Lab (version 6.1.0), RStudio (version 1.4.1717), MaxQuant (version 2.4.12.0), MSFragger (version 22.0).

For manuscripts utilizing custom algorithms or software that are central to the research but not yet described in published literature, software must be made available to editors and reviewers. We strongly encourage code deposition in a community repository (e.g. GitHub). See the Nature Portfolio guidelines for submitting code & software for further information.

Data

Policy information about availability of data

All manuscripts must include a data availability statement. This statement should provide the following information, where applicable:

- Accession codes, unique identifiers, or web links for publicly available datasets
- A description of any restrictions on data availability
- For clinical datasets or third party data, please ensure that the statement adheres to our policy

Mass spectrometry data have been deposited in the ProteomeXchange Consortium (http://proteomecentral.proteomexchange.org) with the dataset identifier ProteomeXchange: PXD058858.
Project Name: Serine ADP-ribosylation on histones and PARP1 is a cellular target for ester-linked ubiquitylation
Project DOI: Not applicable
Reviewer account details:
Username: reviewer_pxd058858@ebi.ac.uk
Password: Vexi8NUp3bih.
Source data are provided with this paper.
The human proteome used in this study is from UniProt reference proteome UP000005640.

Research involving human participants, their data, or biological material

Policy information about studies with human participants or human data. See also policy information about sex, gender (identity/presentation), and sexual orientation and race, ethnicity and racism.

Reporting on sex and gender	n/a
Reporting on race, ethnicity, or other socially relevant groupings	n/a
Population characteristics	n/a
Recruitment	n/a
Ethics oversight	n/a

Note that full information on the approval of the study protocol must also be provided in the manuscript.

Field-specific reporting

Please select the one below that is the best fit for your research. If you are not sure, read the appropriate sections before making your selection.

☒ Life sciences ☐ Behavioural & social sciences ☐ Ecological, evolutionary & environmental sciences

For a reference copy of the document with all sections, see nature.com/documents/nr-reporting-summary-flat.pdf

Life sciences study design

All studies must disclose on these points even when the disclosure is negative.

Sample size	No statistical methods were used to determine sample size, which was chosen in line with what is the standard of the field. Two to four independent replicates were performed for each experiment to confirm reproducibility according to common practices in the field (2-4 biological replicates). For both quantitative (minimum of 3 replicates) and qualitative experiments (identification of sites; minimum of 2 replicates), statistically significant and conclusive results were obtained with the chosen sample sizes. Our sample size is comparable to, or larger than, those in prior published work.
Data exclusions	No data were excluded.
Replication	Two to four independent replicates were performed for each experiment to confirm reproducibility, all observations were found to be reproducible.
Randomization	Samples were not divided into experimental groups, all replicates for all individual experiments were simultaneously prepared and processed.
Blinding	All samples related to the same experiment were handled simultaneously. During handling, samples were clearly labeled (and thus not blinded), but were however processed in random order to avoid bias. During MS data acquisition samples were clearly labeled and thus not blinded. All data analysis was performed with unbiased software in an unsupervised manner and all samples of the same experiment were analysed in exactly the same manner.

Reporting for specific materials, systems and methods

We require information from authors about some types of materials, experimental systems and methods used in many studies. Here, indicate whether each material, system or method listed is relevant to your study. If you are not sure if a list item applies to your research, read the appropriate section before selecting a response.

Materials & experimental systems

n/a	Involved in the study
☐	☒ Antibodies
☐	☒ Eukaryotic cell lines
☒	☐ Palaeontology and archaeology
☒	☐ Animals and other organisms
☒	☐ Clinical data
☒	☐ Dual use research of concern
☒	☐ Plants

Methods

n/a	Involved in the study
☒	☐ ChIP-seq
☒	☐ Flow cytometry
☒	☐ MRI-based neuroimaging

Antibodies

Antibodies used

The following primary antibodies were used for immunoblotting:
Anti-PARP1 polyclonal antibody, Abcam, Cat # ab32138
Anti-Mono-ADP-ribose, clone AbD43647, Bio-Rad ,Cat # TZA020
anti-GFP antibody, clone JL-8, Takara, Cat # 632381
Anti-H3 polyclonal antibody ,Cell Signaling Technology, Cat # 9715S
Anti-ADPRHL2 (ARH3) polyclonal antibody, Merck, Cat # HPA027141
Anti-Histone H2B, clone D2H6, Cell Signaling Technology, 12364
HRP-coupled anti-Histone H3, clone D1H2, Cell Signaling Technology, 12648
Anti-Ubiquitin Antibody, clone VU-1, Life Sensors, VU101
The following secondary antibodies were used:
Anti-mouse IgG HRP-conjugated secondary, Amersham, Cat # NA931V,
Anti-rabbit IgG HRP-conjugated secondary, Merck, Cat # GENA934-1ML.

Validation

All commercial antibodies were validated by manufacturers for the use in immunoblotting.

Anti-PARP1 polyclonal antibody was validated by the manufacturer for use in western blotting and reacts with human samples (https://www.abcam.com/en-de/products/primary-antibodies/parp1-antibody-e102-ab32138#), and additionally validated by us with PARP1KO cells (Bonfiglio et al., Cell, 2020).

The anti-mono-ADPr antibody AbD43647 was extensively validated in previous published papers for immunoblotting and immunofluorescence, among other applications (Bonfiglio et al., Cell, 2020; Longarini et al., Mol Cell, 2023).

Anti-GFP antibody is validated by Takara for the use in western blotting (https://www.takarabio.com/documents/Certificate%20of%20Analysis/632380/632380-632381-070313.pdf).

Anti-H3 polyclonal antibody was validated by the manufacturer for the use in western blotting on a variety of cell lines (https://www.cellsignal.com/products/primary-antibodies/histone-h3-antibody/9715).

Anti-ADPRHL2 (ARH3) antibody was validated by the manufacturer for use in western blotting (https://www.sigmaaldrich.com/DE/de/product/sigma/hpa027141?srsltid=AfmBOoq7_0ASjSB0qmFugfREfS4YJaCkhuIDLb4SrTNO3N_uxufrclq6) and additionally validated by us with ARH3KO cells (Bonfiglio et al. Cell, 2020).

Anti-Histone H2B (D2H6) (Cell Signaling Technology, 12364) is validated by the manufacturer for the use in western blotting on a variety of cell lines (https://www.cellsignal.com/products/primary-antibodies/histone-h2b-d2h6-rabbit-mab/12364).

HRP-coupled anti-Histone H3 (D1H2) (Cell Signaling Technology, 12648) is validated by the manufacturer for the use in western blotting on a variety of cell lines (https://www.cellsignal.com/products/antibody-conjugates/histone-h3-d1h2-xp-rabbit-mab-hrp-conjugate/12648).

Anti-Ubiquitin Antibody clone VU-1 (Life Sensors, VU101) is validated by the manufacturer for the use in wester blotting on a variety of cell lines (https://lifesensors.com/product/vu101-anti-ubiquitin-antibody-mab-clone-vu-1/).

Eukaryotic cell lines

Policy information about cell lines and Sex and Gender in Research

Cell line source(s)

U2OS WT cell lines were obtained from ATCC. U2OS doxycycline inducible GFP-RNF114 WT and GF-RNF114 C176A cell lines were generated as described in the material and methods. ARH3 KO U2OS cells were generously provided by Ivan Ahel

	(University of Oxford).
Authentication	U2OS cell lines were obtained, authenticated by STR profiling and confirmed mycoplasma free by ATCC cell line authentication services.
Mycoplasma contamination	Cells were routinely tested for mycoplasma contamination and confirmed mycoplasma negative.
Commonly misidentified lines (See ICLAC register)	No commonly misidentified lines were used.

Plants

Seed stocks	n/a
Novel plant genotypes	n/a
Authentication	n/a
