# Supplementary material for: Serine ADPr on histones and PARP1 is a cellular target of ester-linked ubiquitylation
Source: Nat Chem Biol. 2025 Jul 9;21(11):1762–72. doi: 10.1038/s41589-025-01974-5 (PMC12568645; doi:10.1038/s41589-025-01974-5)
Supplement: Supplementary file 4 — Unprocessed western blots. [file 41589_2025_1974_MOESM4_ESM.pdf]

Fig. 3c

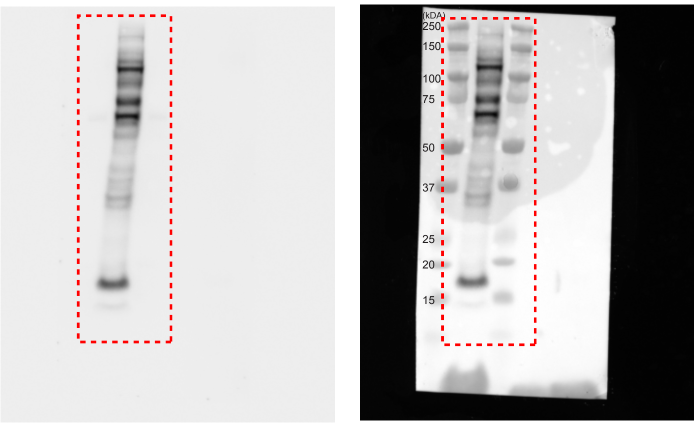

Input
α-mono-ADPr
AbD43647 HRP-coupled

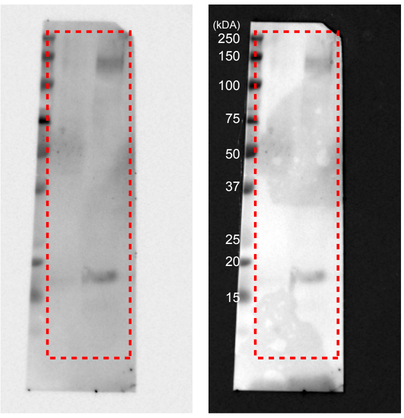

Pulldown of GFP-ZUD
α-mono-ADPr
AbD43647 HRP-coupled

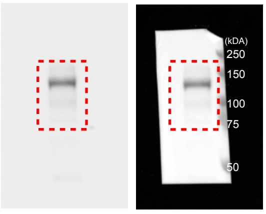

Input
α-PARP1

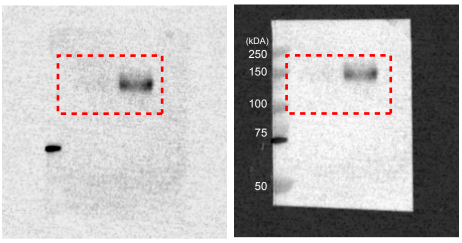

Pulldown of GFP-ZUD
α-PARP1

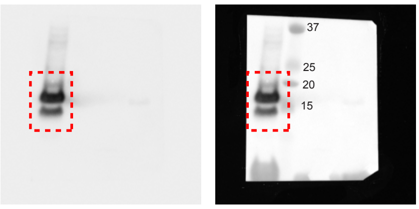

Input
α-H3

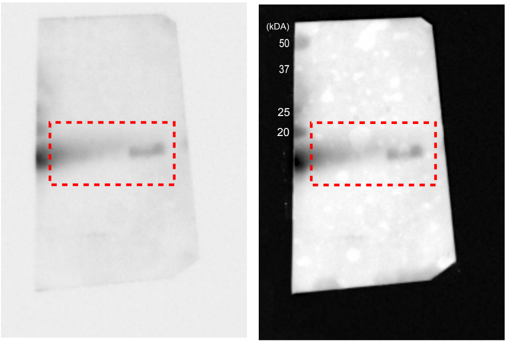

Pulldown of GFP-ZUD
α-H3

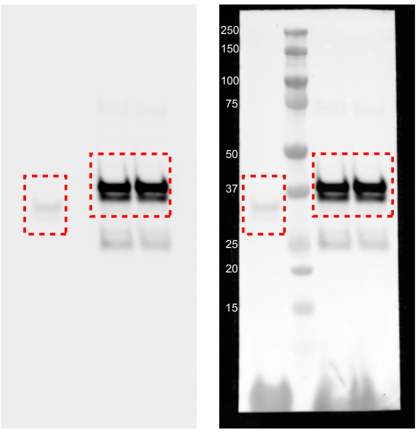

Input
α-GFP

95°C after
EDTA elution
α-GFP

Input
α-GFP

95°C after
EDTA elution
α-GFP
