# Supplementary figures and images for: Serine ADPr on histones and PARP1 is a cellular target of ester-linked ubiquitylation
Source: Nat Chem Biol. 2025 Jul 9;21(11):1762–72. doi: 10.1038/s41589-025-01974-5 (PMC12568645; doi:10.1038/s41589-025-01974-5)

**Fig. 6b**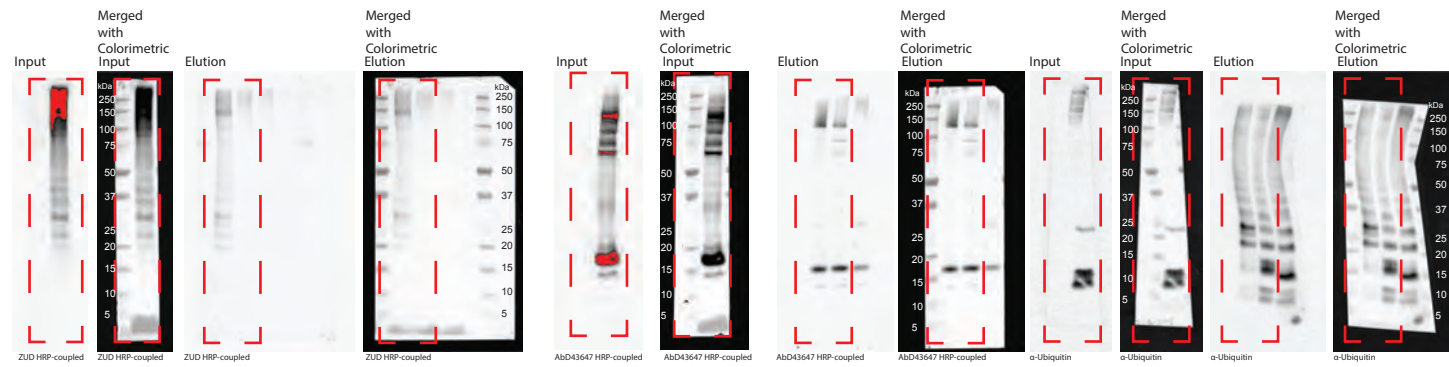**Fig. 6c**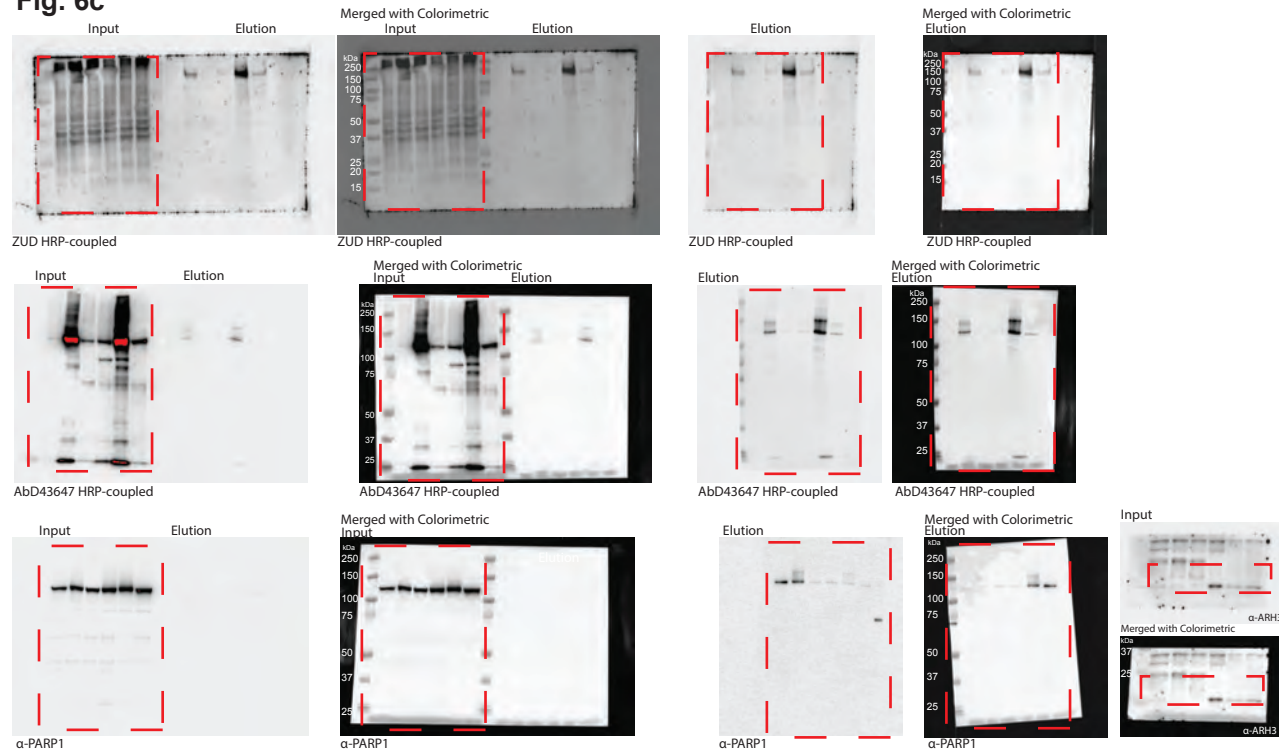**Fig. 6d**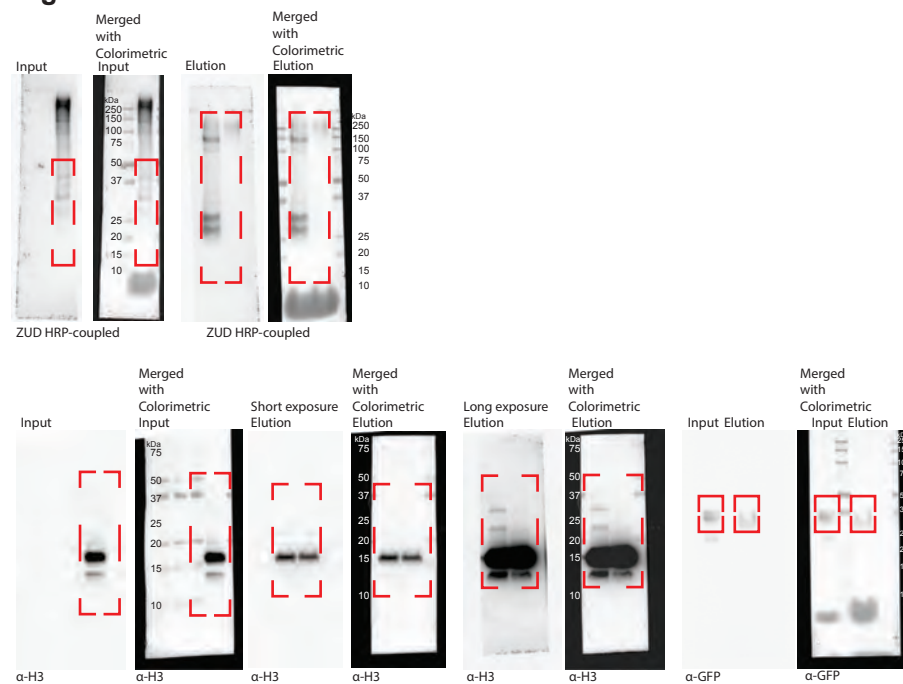

Supplement: Supplementary file 5 — Unprocessed western blots. [file 41589_2025_1974_MOESM5_ESM.pdf]

Extended Data Fig. 1a

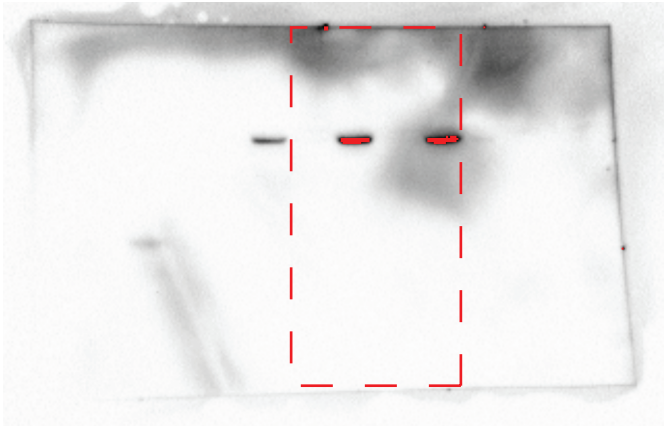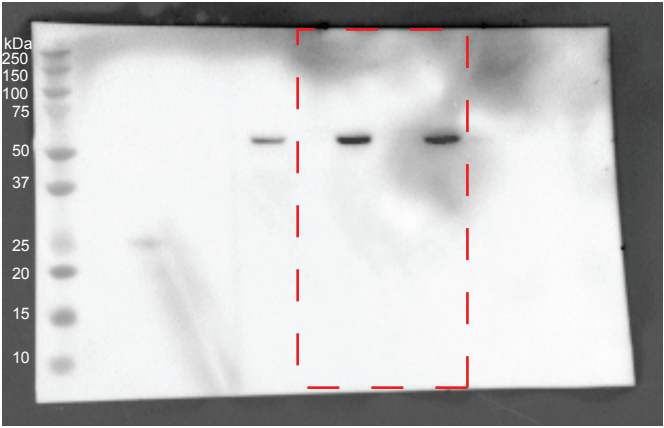

Supplement: Supplementary file 6 — Unprocessed western blots. [file 41589_2025_1974_MOESM6_ESM.pdf]

## Extended Data Fig. 2c

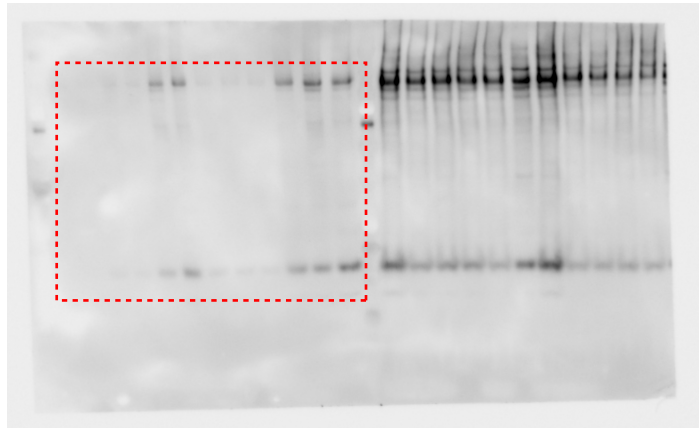

Pulldown of GFP-ZUD  
 $\alpha$ -mono-ADPr  
AbD43647 HRP-coupled

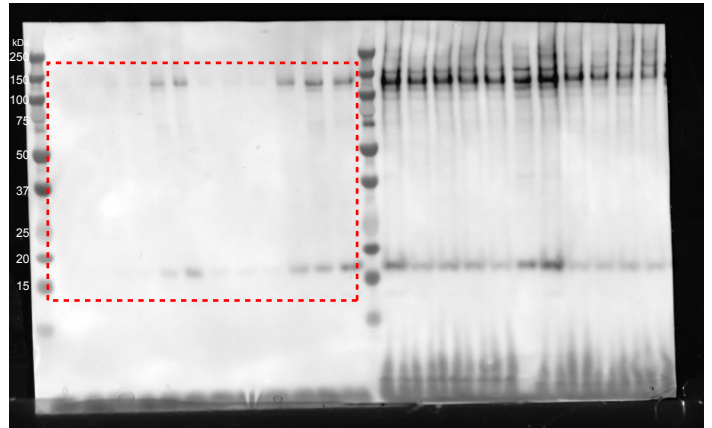

Supplement: Supplementary file 7 — Unprocessed western blots. [file 41589_2025_1974_MOESM7_ESM.pdf]

Extended Data Fig. 6b

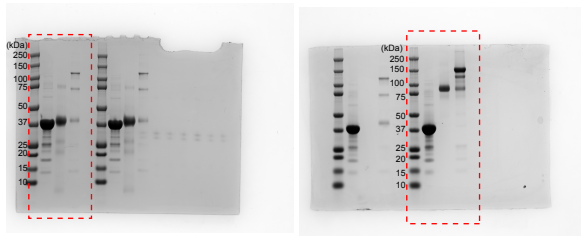

Extended Data Fig. 6c

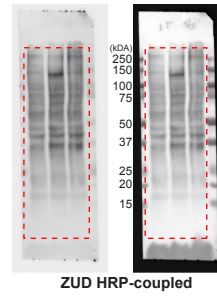

ZUD HRP-coupled

Extended Data Fig. 6d

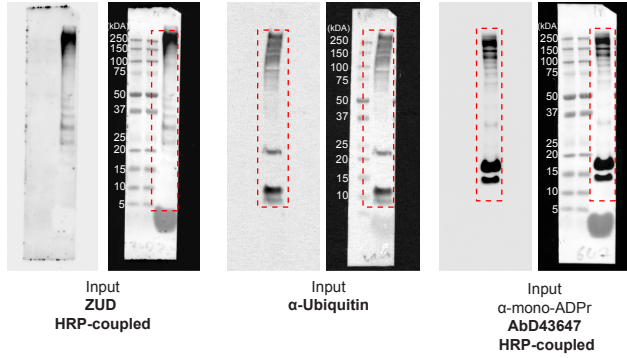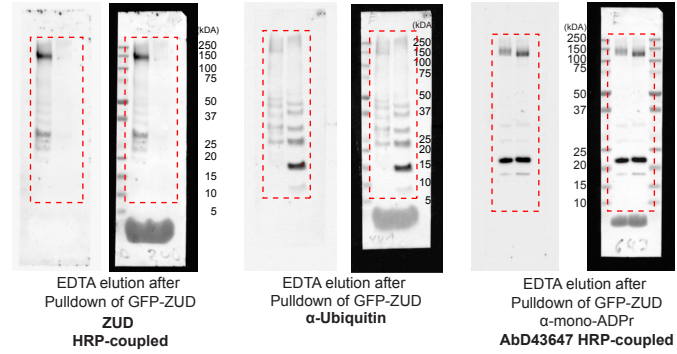

Supplement: Supplementary file 8 — Unprocessed western blots. [file 41589_2025_1974_MOESM8_ESM.pdf]

Extended Data Fig. 7a

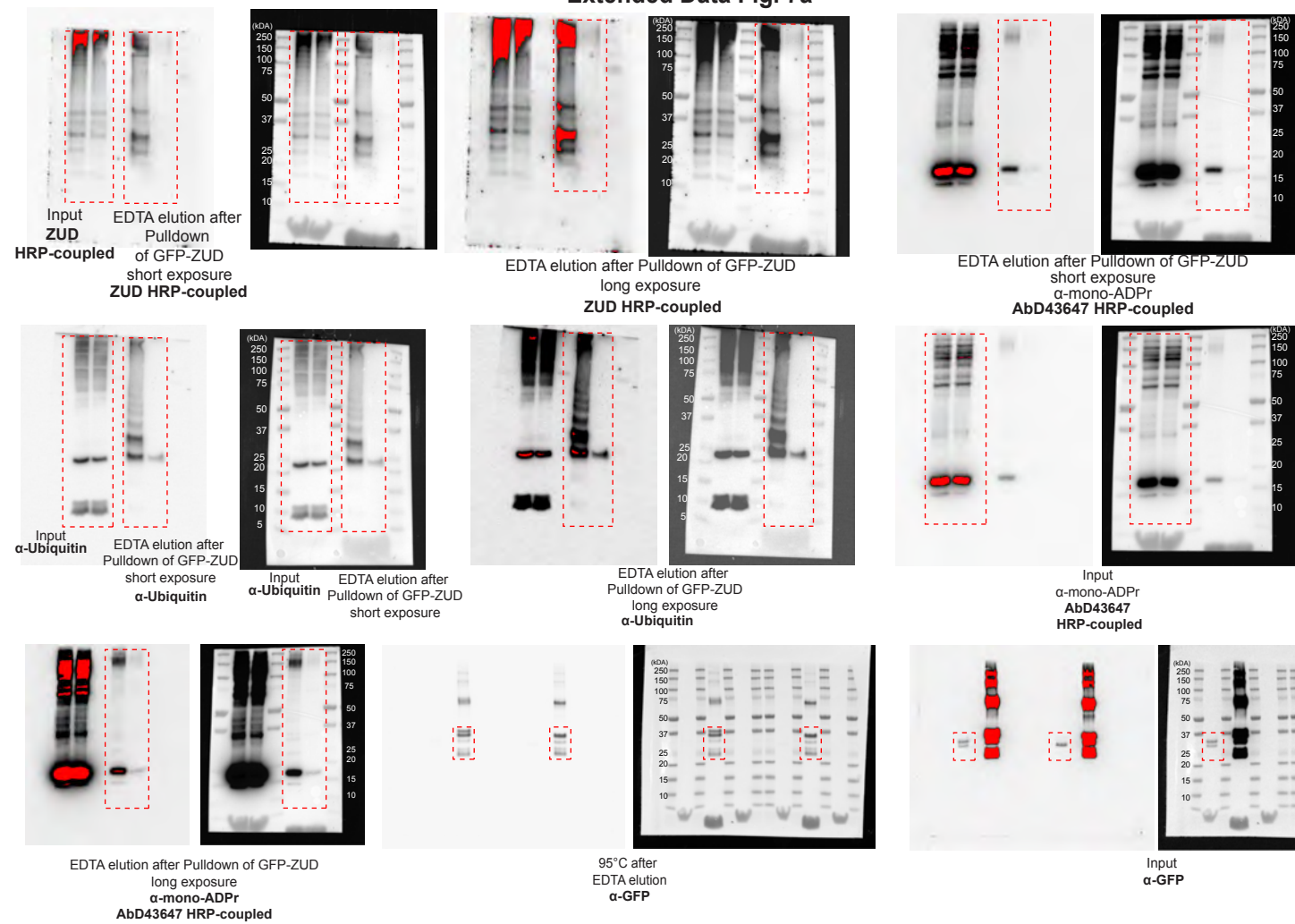

Extended Data Fig. 7b

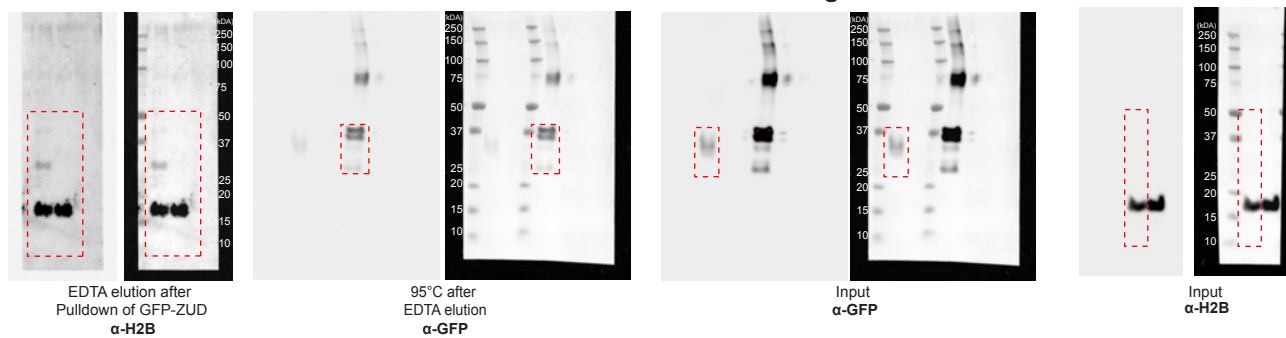

Supplement: Supplementary file 9 — Unprocessed western blots. [file 41589_2025_1974_MOESM9_ESM.pdf]
